# Loss of SOCS1 in Donor T Cells Exacerbates Intestinal GVHD by Driving a Chemokine‐Dependent Pro‐Inflammatory Immune Microenvironment

**DOI:** 10.1002/advs.202513735

**Published:** 2026-01-25

**Authors:** Zhigui Wu, Bixia Wang, Xinya Jiang, Fangqing Zhang, Qianqian Huang, Xinyi Wu, Shuang Fan, Qi Zhang, Jingrui Zhou, Jianing Tang, Xiao‐Dong Mo, Yu Wang, Ying‐Jun Chang, Huidong Guo, Xiao‐Jun Huang

**Affiliations:** ^1^ Peking University People's Hospital Peking University Institute of Hematology National Clinical Research Center for Hematologic Disease Beijing Key Laboratory of Cell and Gene Therapy for Hematologic Malignancies Peking University Beijing China; ^2^ Peking‐Tsinghua Center for Life Sciences Academy for Advanced Interdisciplinary Studies Peking University Beijing China; ^3^ Collaborative Innovation Center of Hematology Peking University Beijing China

**Keywords:** bone marrow transplantation, cell migration, chemokines, gastrointestinal tract, intestinal T cells

## Abstract

Acute graft‐versus‐host disease (aGVHD) of the gastrointestinal tract is a frequent and often fatal complication of allogeneic hematopoietic stem cell transplantation. Suppressor of Cytokine Signaling 1 (SOCS1) is a key regulator of T cell pathogenicity, yet its role in aGVHD remains unclear. Using T cell‐specific *Socs1* knockout models, we show that *Socs1* loss intrinsically drives pro‐inflammatory T cell differentiation independent of antigen stimulation, with the strongest effects observed in CD8^+^ T cells. Mechanistically, *Socs1* deficiency activates a STAT1/2‐dependent transcriptional program, inducing *Ccl5* expression, monocyte recruitment, and M1‐like macrophage polarization in peripheral lymphoid organs at steady state. After transplantation, *Socs1*‐deficient T cells display enhanced infiltration into intestinal crypts, accompanied by increased CD8^+^ T cell effector function, monocyte accumulation, and inflammatory macrophage polarization in target tissues. These changes promote tissue injury and impair regeneration, resulting in lethal aGVHD. Importantly, JAK1/2 inhibition with ruxolitinib reverses these pathogenic effects. Clinically, high *SOCS1* expression in donor‐derived CD8^+^ T cells correlates with reduced aGVHD incidence. Together, our findings identify SOCS1 as a predictive biomarker and a potential therapeutic target for personalized aGVHD prophylaxis.

## Graphical abstract

T cell‐specific *Socs1* knockout leads to inflammatory differentiation of CD8^+^ T cells, prompting the STAT1/2 complex to drive the activation of *Ccl5*, *Ccr5*, and *Cxcr3*, and promoting the skewing of monocytes toward a pro‐inflammatory M1 macrophage lineage. After transplantation, *Socs1*‐deficient CD8^+^ T cells infiltrate the intestine and orchestrate a vicious inflammatory cycle by recruiting monocytes via CCL5 secretion, thereby sustaining crypt‐localized inflammation and exacerbating intestinal injury during aGVHD. Although a CCL5‐neutralizing antibody, Maraviroc, and Ruxolitinib all alleviate intestinal GVHD in *Socs1*‐deficient models, Ruxolitinib demonstrates superior efficacy. Clinically, high *SOCS1* expression in CD8^+^ T cells correlates with reduced aGVHD incidence following allo‐HSCT.

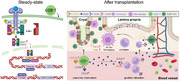

## 1 Introduction

Allogeneic hematopoietic stem cell transplantation (allo‐HSCT) is a curative therapy for a wide range of conditions, including hematological malignancies, severe aplastic anemia, and primary immunodeficiencies such as severe combined immunodeficiency (SCID) [1, 2]. However, aGVHD remains a major and life‐threatening complication, occurring in 30%–50% of patients, with severe cases affecting 14%–36% [3, 4]. While first‐line treatments, such as glucocorticoids, have improved outcomes in some patients, the prognosis for those with severe or refractory aGVHD remains poor, with a 2‐year survival rate below 20% [4]. Alternative therapeutic approaches to reducing aGVHD damage are an unmet medical need.

Acute GVHD primarily targets the skin, gastrointestinal tract, and liver, with the intestinal tract serving as a central site for disease initiation and amplification. Pre‐transplant conditioning disrupts the intestinal mucosa, triggering the release of damage‐ and pathogen‐associated molecular patterns (DAMPs and PAMPs), which drive T cell‐mediated immune activation [5]. This inflammatory response exacerbates intestinal injury and promotes aGVHD progression. During this process, complex interactions between adaptive immune cells, particularly T cells, and innate immune cells, including monocytes and macrophages, play a critical role. Allogeneic macrophages display substantial heterogeneity and plasticity, contributing to diverse aGVHD outcomes. Classically activated M1 macrophages, induced by Th1 cytokines (e.g., IL‐2, IFN‐γ), promote inflammation and tissue damage [6, 7], whereas alternatively activated M2 macrophages, driven by Th2 cytokines (e.g., IL‐4, IL‐10), exhibit anti‐inflammatory and pro‐repair functions [6, 7]. Owing to this functional diversity, macrophages have emerged as both therapeutic targets [8, 9, 10] and potential cell‐based therapies [11, 12] in aGVHD. However, the regulatory mechanisms and temporal dynamics underlying T cell‐macrophage interactions and M1‐M2 polarization in both peripheral and tissue‐resident compartments remain poorly understood under homeostatic and aGVHD conditions.

SOCS1 is a key negative regulator of cytokine‐driven T cell activation through inhibition of the JAK/STAT pathway. Global *Socs1* deficiency causes neonatal lethality, which can be rescued by either genetic deletion of *Ifng* or IFN‐γ neutralization [13, 14]. While systemic loss of *Socs1* leads to fatal inflammation, T cell‐specific deficiency does not induce lethal inflammatory pathology [15, 16]. In T cells, *SOCS1* expression is induced by cytokines such as IFN‐γ and signaling through the common IL‐2 receptor (IL‐2Rγc), with its impact on T cell polarization being context dependent [17, 18, 19, 20]. Our previous work demonstrated that G‐CSF promotes *SOCS1* upregulation through chromatin remodeling in T cells, contributing to T cell hyporesponsiveness [21]. Conditional deletion of *Socs1* in T cells not only exacerbated aGVHD severity but also abrogated the protective effects of G‐CSF in murine models [21]. The central role of SOCS1 in restraining the JAK/STAT pathway highlights this axis as a key therapeutic target in aGVHD. Indeed, the JAK1/2 inhibitor ruxolitinib [22] has been approved for the treatment of steroid‐refractory aGVHD, underscoring the clinical relevance of this pathway. However, the downstream mechanisms by which *SOCS1* levels within T cells dictate the inflammatory landscape and confer protection against aGVHD remain incompletely understood.

In the present study, we systematically investigated the regulatory function of SOCS1 within T cells during the pathogenesis of gastrointestinal aGVHD. By leveraging T‐cell‐specific *Socs1* knockout models coupled with multi‐omics profiling, we sought to delineate the molecular programs driving tissue‐specific immunopathology. Our study reveals that *Socs1* acts as a critical checkpoint that not only restrains the intrinsic pathogenic potential of CD8^+^ T cells but also orchestrates the broader immune microenvironment to preserve intestinal integrity. These findings highlight the fundamental importance of intrinsic negative regulation in mitigating aGVHD and suggest that targeting the SOCS1 axis represents a viable strategy to balance immune activation and tissue tolerance.

## 2 Results

### 2.1 T Cell‐Specific *Socs1* Knockout Leads to Inflammatory Differentiation of T Cells

**FIGURE 1:**
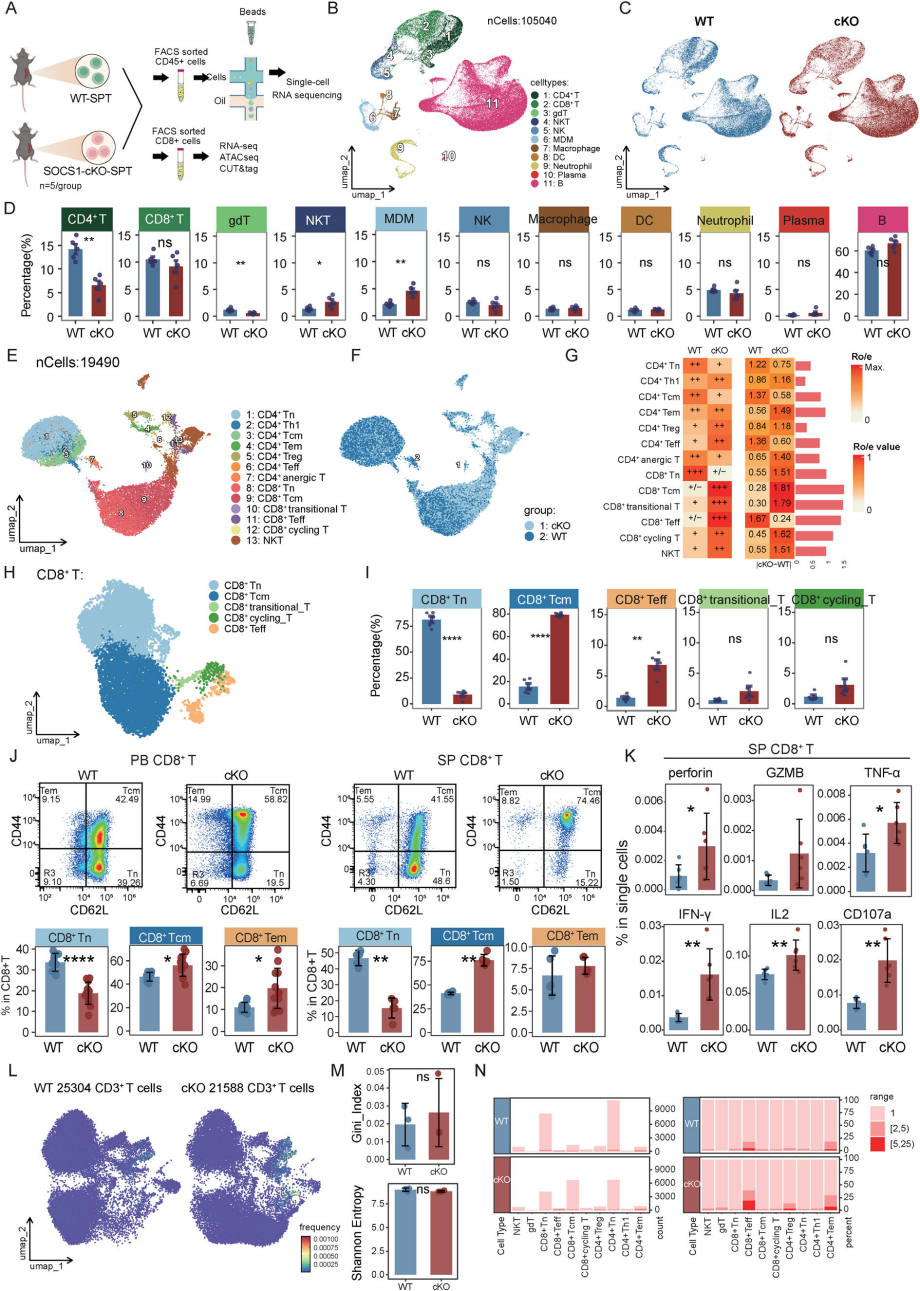
Socs1 deficiency in T cells drives effector differentiation and enhances inflammatory responses in CD8^+^ T cells. (A) Experimental schematic. Splenocytes from WT (littermate control; *Socs1^fl/fl^*) and cKO (LckCre‐*Socs1^fl/fl^*) mice were isolated and sorted by FACS for CD45^+^ cells and subjected to single‐cell RNA sequencing (scRNA‐seq, n = 5 per group). Alternatively, CD8^+^ T cells from the spleen of WT and cKO mice were sorted by FACS and subjected to bulk RNA‐seq, ATAC‐seq, and CUT&Tag analyses. (B) UMAP plot of 105 040 single cells from CD45^+^ splenocytes colored by annotated immune cell subsets. (C) UMAP visualization of CD45^+^ splenocytes, split by origin. (D) Comparison of the proportions of celltypes between WT and cKO groups. (E) UMAP plot of 19 490 T cells extracted from Figure 1B and colored by annotated T cell subsets. (F) UMAP visualization of T cells, split by origin. (G) Heatmap of Ro/e (Ratio of observed to expected) scores for T cell subtypes in WT and cKO mice. The scores, calculated from scRNA‐seq cell counts, indicate the relative enrichment (red, Ro/e > 1) or depletion (white/light orange, Ro/e < 1) of each population within each genotype. Numerical values are presented alongside a semi‐quantitative summary. (H) UMAP plot of 9159 CD8^+^ T cells extracted from Figure 1E and colored by annotated T cell subsets. (I) Comparison of the proportions of indicated CD8^+^ T cell clusters between WT and cKO groups. (J) Representative flow cytometry plots and frequencies of naive T cells (Tn; CD44^−^CD62L^+^), central memory (Tcm; CD44^+^CD62L^+^), and effector memory (Tem; CD44^+^CD62L^−^) in CD8^+^ T cells from peripheral blood (PB, left panel) and spleen (SP, right panel) (n = 5 per group). (K) Bar plots showing the expression of perforin, GZMB, TNF‐α, IFN‐γ, IL‐2, and CD107a in CD8^+^ T cells from WT and cKO mice, as measured by flow cytometry (n=5 per group). (L) UMAP visualization of integrated T‐cell transcriptomes from WT (left, 25 304 cells) and cKO (right, 21 588 cells) groups. Each point represents a single cell, colored by the frequency of its corresponding TCR clonotype, highlighting clonally expanded cells. (M) Quantification of overall TCR repertoire diversity. The Gini index (top) and Shannon entropy (bottom) were calculated for the entire T‐cell population from each mouse. (N) Distribution and clonal size of T cells across identified subsets. Barplot showing the absolute cell counts (left panels) and the clonal size composition (right panels) for each T‐cell subset from WT and cKO mice. Data represent one experiment out of two independent experiments. *P* values were determined using two‐sided Wilcoxon rank‐sum test (D, I, M) or unpaired two‐tailed Student's t‐test (J‐K). Data represent mean ± SEM (D, I, M) or mean ± SD (J‐K). ^∗^ *p* <.05, ^∗∗^ *p* <.01 and ^∗∗∗∗^ *p* <.0001.

To investigate the immune profile dynamics resulting from T cell‐specific SOCS1 deletion, we sorted CD45^+^ splenocytes from wild‐type (WT, Socs1^fl/fl^) and conditional knockout (cKO, LckCre‐Socs1^fl/fl^) mice and subjected them to single‐cell RNA sequencing using the 10× Genomics platform (Figure 1A). After clustering, a total of 105 040 cells from both groups were grouped into 11 clusters, including CD4^+^ T, CD8^+^ T, γδT, NKT, NK, MDM (Monocyte‐Derived Macrophage), Macrophage, DC, Neutrophil, Plasma, and B cells (Figure 1B,C; Figure S1A). Consistent with a previous study [23], we observed a significant reduction in the proportion of CD4^+^ T cells in the cKO group compared to WT mice, while the proportion of CD8^+^ T cells remained unchanged. In contrast, the MDM and NKT cell populations were significantly expanded in the cKO group (Figure 1D). A detailed subclustering of all T cells (except for γδT cells) resolved them into 11 distinct populations (Figure 1E,F; Figure S1B). Of these, the CD8^+^ T cell lineage showed the greatest alterations in proportional representation between the two groups, as determined by Ro/e analysis (Figure 1G). Interestingly, although the overall proportion of CD8^+^ T cells was not significantly affected by *Socs1* deletion, their subset composition was dramatically altered (Figure 1H,I; Figure S1B). Specifically, cKO mice exhibited a significant decrease in naïve CD8^+^ T cells (Tn) and a concomitant increase in both central memory (Tcm) and effector (Teff) CD8^+^ T cell populations compared to WT controls (Figure 1I). These changes were further validated by flow cytometry. The reduction in Tn cells and the increased Tcm and Tem cells were consistently observed in CD8^+^ T cells from both peripheral blood (PB) and spleen (SP) of cKO mice (Figure 1J). Moreover, CD8^+^ T cells in the spleen of cKO mice exhibited enhanced cytotoxicity, as evidenced by elevated expression of perforin, granzyme B (GZMB), TNF‐α, IFN‐γ, IL‐2, and CD107a (Figure 1K).

Furthermore, despite their reduced overall proportion in the cKO group (Figure 1D), CD4^+^ T cells underwent significant compositional shifts, with a decrease in naïve/central memory subsets and an expansion of effector memory, terminal effector, and Treg populations (Figure S1C,D). This was underpinned at the transcriptomic level by the broad upregulation of pro‐inflammatory and Th1‐associated genes (e.g., *Ifng*, *Gzmb*, *Tbx21*, *Stat1*) in bulk cKO CD4^+^ T cells (Figure S1E). This Th1‐like program also infiltrated the Treg compartment (Figure S1F). cKO Tregs maintained stable Foxp3 expression but co‐expressed *T‐bet* and *Stat1*, identifying them as pathogenic “Th1‐like Tregs” [24].

Given the increased proportion of NKT cells observed in *Socs1*‐deficient mice, we next examined NKT cell subset characteristics. Differential gene expression analysis revealed that NKT cells from the cKO group exhibited a highly pro‐inflammatory and cytotoxic transcriptional profile, with upregulation of genes including *H2‐Q7*, *Gzma*, *Gzmb*, *Ccl5*, *Klrg1*, *Nkg7*, *Tbx21*, and *Ifng* (Figure S1G). To identify the cellular source of this shift, we further classified NKT cells into the three canonical subsets, iNKT0, iNKT1, and iNKT2/17, based on established markers [25, 26] (Figure S1H,I). This analysis demonstrated a dramatic enrichment of the pathogenic iNKT1 subset specifically within the cKO group. Importantly, previous work by Kristina Maas‐Bauer et al. [26] demonstrated that iNKT cell subsets are functionally heterogeneous in GVHD, with iNKT2 and iNKT17, but not iNKT1, conferring a significant survival benefit. Together, these findings indicate that *Socs1* deficiency skews the T cell compartment toward a pro‐inflammatory immune state even in the absence of antigenic stimulation.

To define the impact of *Socs1* deficiency on the T‐cell repertoire at a single‐cell level, we performed scTCRseq on splenic CD3^+^ T cells from WT and cKO mice. A total of 25 304 T cells from WT and 21 588 T cells from cKO mice were included in the final analysis (Figure 1L). While UMAP visualization showed the presence of expanded clonotypes in both groups (Figure 1L), a quantitative analysis of the entire TCR repertoire revealed no significant differences in overall diversity or clonality, as measured by the Shannon entropy and Gini index, respectively (Figure 1M). However, compared to WT mice, the cKO group exhibited a slight increase in the absolute number of CD8^+^ effector T cells (Figure 1N, left panel). While nearly all T‐cell subsets in WT mice were composed of unique clonotypes, a significant proportion of the CD8^+^ Teff population in cKO mice consisted of expanded clones (clonal size >2), indicating a targeted proliferation of effector T cells (Figure 1N, right panel).

Collectively, these findings suggest that Socs1 deficiency drives a substantial bias in T cell differentiation and activation, with this effect being most prominent in CD8^+^ T cells, which are pushed toward a more inflammatory and cytotoxic phenotype.

### 2.2 STAT1/2 Complex Drives Activation of *Ccl5*, *Ccr5*, and *Cxcr3* in *Socs1*‐Deficient CD8^+^ T Cells

**FIGURE 2:**
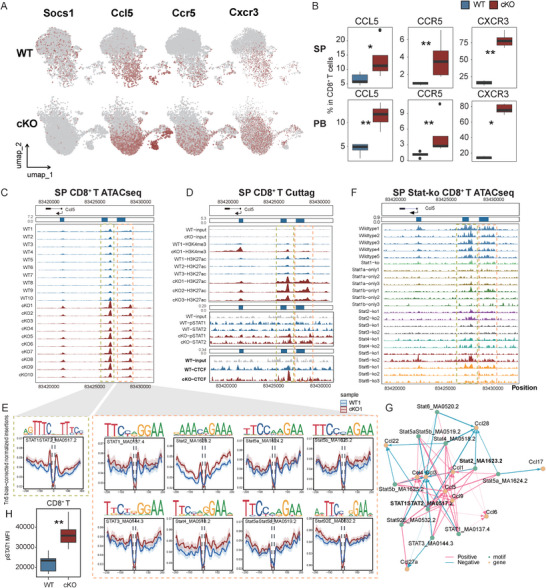
Chromatin remodeling and transcription factor binding promote upregulation of *Ccl5, Ccr5, and Cxcr3* in Socs1‐deficient CD8^+^ T cells. (A) UMAP plots illustrating the expression of *Socs1, Ccl5, Ccr5*, and *Cxcr3* in CD8^+^ T cells from scRNA‐seq data in Figure 1 between WT and cKO groups. (B) Boxplots showing the expression of *CCL5, CCR5*, and *CXCR3* in CD8^+^ T cells from SP (top) and PB (bottom), measured by flow cytometry (n = 5 per group). (C) ATAC‐seq signal tracks at the *Ccl5* gene region (chr11:83,420,603–83,430,255, mm39) in splenic CD8^+^ T cells from WT and cKO mice. Signal intensities represent normalized read coverage and were visualized using the trackVis function with a fixed *y*‐axis range from 0 to 7.2 across all samples to ensure consistency in comparison. Gene model annotations and genomic coordinates are displayed above the tracks. (D) CUT&Tag signal tracks showing chromatin features at the *Ccl5* locus (same genomic coordinates) in CD8^+^ T cells from WT and cKO mice. Data include antibody‐specific signals for H3K27ac, H3K4me3, input, CTCF, pSTAT1, and STAT2. *Y*‐axis signal ranges are indicated on the left (e.g., 0.0–5.3) and were set per group to optimize peak visualization. (E) Footprint plots illustrating Tn5 accessibility patterns in various STAT‐related DNA motifs located at the *Ccl5* locus in CD8^+^ T cells from WT1 and cKO1 mice. (F) ATAC‐seq signal tracks from the GSE204736 dataset showing chromatin accessibility at the Ccl5 locus in splenic CD8^+^ T cells from WT and various Stat knockout mice, with the *y*‐axis signal range (0.0–0.9) indicated on the left of the tracks for reference. (G) Gene regulatory network showing positive (pink) and negative (blue) correlations between transcription factor motif binding signal and the expression levels of associated genes. Green nodes represent TF motifs and orange nodes represent genes. Edge direction indicates correlation direction, and line thickness reflects correlation strength. (H) Boxplot showing the expression (MFI) of pSTAT1(Ser727) in CD8^+^ T cells isolated from the spleen of WT and cKO mice and measured by flow cytometry (n = 5 per group). Data represent one experiment out of two independent experiments. *P* values were determined using an unpaired two‐tailed Student's t‐test (B, H). ^∗^ *p* <.05 and ^∗∗^ *p* <.01.

To further investigate transcriptomic and epigenomic alterations in *Socs1*‐deficient CD8^+^ T cells, we sorted CD8^+^ T cells from the spleens of WT and cKO mice and performed RNA‐seq, ATAC‐seq, and CUT&Tag analyses (Figure 1A; Data S1–S3). *Socs1*‐deficient CD8^+^ T cells exhibited marked upregulation and increased chromatin accessibility of key chemokine‐related genes, including *Ccl5*, *Ccr5*, and *Cxcr3* (Figure 2A; Figure S2A, Data S4). Notably, this pattern was also observed in CD8^+^ T cells from patients with aGVHD [27], where expression of *CCL5*, *CCR5*, and *CXCR3* was significantly higher compared to healthy controls (Figure 2A; Figure S2A). Flow cytometry further confirmed elevated protein levels of CCL5, CCR5, and CXCR3 in *Socs1*‐deficient CD8^+^ T cells from both PB and spleen (Figure 2B). Globally, cKO triggered a strong induction of *Ccl5* across the T cell compartment, with elevated expression observed in all differentiation‐derived subsets. Among these, CD8^+^ Tcm cells emerged as the major *Ccl5*‐producing population (Figure S2B,C). Together, the coordinated upregulation of *Ccl5* and its associated chemokine receptors is expected to promote the trafficking of activated CD8^+^ T cells to systemic tissues [28, 29, 30, 31]. Within these tissues, CCL5 secreted by activated CD8^+^ T cells may further facilitate the recruitment of additional immune cell populations.

ATAC‐seq analysis revealed significantly increased chromatin accessibility at two loci (Peak 1 positions 83,426,035; Peak 2 83,428,088) upstream of the transcription start site (TSS) of *Ccl5* in *Socs1*‐deficient CD8^+^ T cells (Figure 2C). Notably, Peak 1 overlaps with a previously defined proximal enhancer of *Ccl5* [32]. Integrated analysis of CUT&Tag and Hi‐C data further demonstrated a marked increase in H3K27ac signals at both Peak 1 and Peak 2 in CD8^+^ T cells from cKO mice. These two enhancer regions were found to form loop structures with the *Ccl5* promoter, suggesting enhanced enhancer‐promoter interactions that likely contribute to the elevated transcription of *Ccl5* in *Socs1*‐deficient CD8^+^ T cells (Figure 2D; Figure S2D).

Given that SOCS1 is a known negative regulator of JAK/STAT signaling, we next investigated which STAT proteins are involved in the transcriptional activation of *Ccl5*. Footprint analysis of STAT binding at the two enhancer loci revealed that the STAT1:STAT2 heterodimer binds to Peak 1, while multiple STAT proteins bind to Peak 2 (Figure 2E). Supporting this, CUT&Tag profiling revealed an increase in pSTAT1 occupancy at Peak 1 in *Socs1*‐deficient CD8^+^ T cells, with no notable change at Peak 2. In contrast, STAT2 binding was markedly elevated at both Peak 1 and Peak 2 in the cKO group (Figure 2D), indicating a potential role for both STAT1 and STAT2 in regulating *Ccl5* expression. To further validate these findings, we analyzed the public dataset (GSE204736 [33]). Knockout of *Stat1* (either partially or completely), as well as *Stat2*, *Stat3*, *Stat4*, *Stat5*, and *Stat6*, all led to reduced chromatin accessibility at the Peak 1 and Peak 2 regions (Figure 2F), implicating multiple STATs in *Ccl5* regulation. Notably, interaction analysis identified the STAT1/STAT2 complex as the strongest positive regulator of *Ccl5* (Figure 2G). Flow cytometry further confirmed increased phosphorylation of STAT1 at Ser727 in CD8^+^ T cells from cKO mice, supporting its enhanced transcriptional activity (Figure 2H; Figure S2E). Furthermore, we observed increased chromatin accessibility at the promoters of *Ccr5* and *Cxcr3* in cKO cells, evidenced by enhanced openness at H3K4me3‐ and H3K27ac‐enriched regions (Figure S2F,G). Together, these findings suggest that SOCS1 suppresses *Ccl5* transcription by inhibiting STAT1 and STAT2 activity. In the absence of SOCS1, the STAT1/STAT2 complex mediates enhancer‐promoter interactions at the *Ccl5* loci, thereby promoting *Ccl5* expression. The concurrent upregulation of *Ccl5, Ccr5*, and *Cxcr3* may further enhance CD8^+^ T cells activation and migration.

### 2.3 *Socs1*‐Deficient CD8^+^ T Cells Drive Monocyte Polarization Toward M1 Macrophages

**FIGURE 3:**
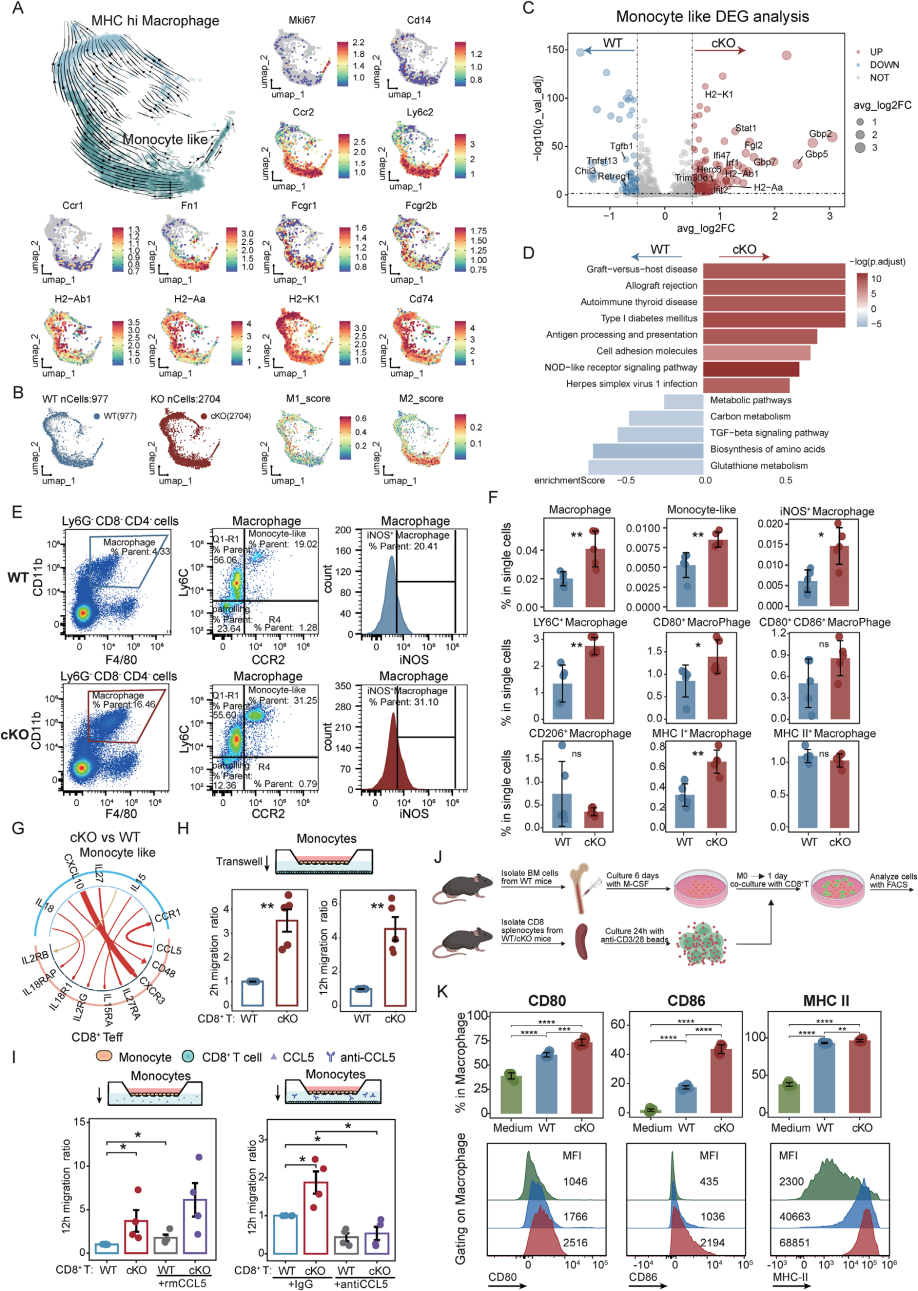
*Socs1* cKO Drives Monocyte Polarization Toward M1 Macrophages in Mice. (A) UMAP plot of annotated subsets extracted from MDM cells based on scRNA‐seq data in Figure 1, colored by clusters, and integrated with RNA velocity analysis to illustrate predicted future transcriptional states and developmental trajectories of the subsets. UMAP plots of feature genes are displayed around the main plot. (B) UMAP plots showing the distribution of MDM cells in WT and cKO groups, and the visualization of M1 and M2 signature scores. (C) Differential expression genes (DEGs) analysis of Monocyte‐like cells between WT and cKO groups. Red indicated upregulation in the cKO group, and blue indicated downregulation in the cKO group. (D) KEGG pathway analysis based on DEGs in Figure 3C. (E) Representative flow cytometry plots and frequencies of F4/80^low^CD11b^hi^ Macrophage, Monocyte‐like cells, and iNOS^+^ Macrophage in the spleen of WT and cKO mice (n = 5 per group). (F) Quantification of macrophage subpopulations in WT and cKO mice. Bar plots show the frequency (% of total single cells) of the indicated macrophage populations in the spleen as determined by flow cytometry. Populations shown include total macrophages, monocyte‐like, iNOS^+^, Ly6C^+^, CD80^+^, CD80^+^, CD86^+^, CD206^+^, MHC class I^+^ and MHC class II^+^ macrophages. (G) iTALK‐identified and visualized ligand‐receptor interaction signals between Monocyte‐like subsets and CD8^+^ Teff subsets, illustrating differential intercellular communication patterns in the cKO group compared to the WT group. Legend is shown in Figure S3G. (H) Transwell migration assay showing the migration rate of monocytes from WT mice toward CD8^+^ T cells derived from WT or cKO mice after 2 h (left) and 12 h (right) (n = 5). (I) Transwell migration assay showing the 12‐h migration rate of monocytes from WT mice toward CD8^+^ T cells derived from WT or cKO mice, either with or without CCL5 activation (rmCCL5, left panel), and with CCL5 neutralization using an anti‐CCL5 antibody (right panel) (n=4). (J) Experimental schematic showing the co‐culture system of BMDMs and CD8^+^ T cells. (K) Barplot and flow cytometry histogram showing expression of activation markers (CD80, CD86) and MHC Class II on macrophages following co‐culture with T cells from two groups. Data represent one experiment out of two independent experiments. *P* values were determined using an unpaired two‐tailed Student's t‐test (F, H‐I, K). Data represent mean ± SD (F, H‐I, K). ^∗^∗P <.05, ∗∗P <.01 and ∗∗∗∗P <.0001.

T cell‐specific deletion of *Socs1* reshaped the immune microenvironment in lymphoid organs, notably increasing MDMs in the spleen of cKO mice (Figure 1D). To investigate further, we analyzed 3681 MDMs using RNA velocity, revealing a differentiation trajectory from monocyte‐like cells to MHC^hi^ macrophages, with cKO cells enriched at the terminal stage (Figure 3A,B; Figure S3A). Early‐stage monocyte‐like cells exhibited monocyte‐associated genes (*Cd14*, *Ccr2*, *Ly6c2*, *Ccr1*) along with M2 macrophage‐associated markers (*Siglec1*, *Mrc1*, *Trem2*). In contrast, terminal‐stage MHC^hi^ macrophages expressed M1‐associated signatures (*FcgR1*, *H2‐Aa*, *H2‐Ab1*, *H2‐K1*, *Cd74*) [34, 35]. (Figure 3A,B; Figure S3A). Consistently, cKO mice exhibited elevated M1 scores, whereas WT mice showed higher M2 scores (Figure S3B). cKO monocyte‐like cells also demonstrated a pro‐inflammatory profile, marked by upregulated interferon‐stimulated genes (*Stat1*, *Ifit2*, *Gbp2/5/7*), elevated MHC class I/II molecules expression (*H2‐K1, H2‐Aa, H2‐Ab1*), and enriched immune activation pathways (Figure 3C,D). In contrast, anti‐inflammatory genes (*Chil3, Tgfb1, Tnfsf13, Trim30d*) and TGF‐β signaling were downregulated (Figure 3C,D). Flow cytometry revealed a significant expansion of the F4/80^low^CD11b^hi^ macrophage [36] population in cKO mice compared to WT controls (Figure 3E,F; Figure S3C). Further characterization demonstrated that these macrophages were strongly skewed toward a pro‐inflammatory, M1‐like phenotype. Specifically, macrophages from cKO mice contained a significantly higher proportion of monocyte‐like cells and displayed increased expression of the canonical M1 marker iNOS. Moreover, they showed significant upregulation of the co‐stimulatory molecule CD80 and the antigen‐presenting molecule MHC Class I (Figure 3F). In contrast, the frequency of M2‐like macrophages, identified by CD206 expression, was not increased in the cKO group. Furthermore, ELISA analysis of whole spleen lysates indicated that the cKO group had elevated overall levels of IL‐1β, IL‐6, and TNF‐α (Figure S3D).

To further investigate the mechanism underlying M1 macrophage accumulation in the spleen of cKO mice, we used iTALK to analyze cell‐cell interactions and found enhanced communication between CD8^+^ T cells and monocyte‐like macrophages via the CCL5‐CCR1 ligand‐receptor pair in cKO mice compared to WT controls. (Figure 3G). Given the high levels of TNF‐α and IFN‐γ secreted by cKO CD8^+^ T cells (Figure 1C), our findings suggest that these T cells may also produce CCL5, which recruits CCR1^+^ monocytes from the peripheral blood into the spleen and promotes their differentiation into M1 macrophages. To validate this, a Transwell assay was performed: WT bone marrow‐derived monocytes (upper chamber) were co‐cultured with splenic cKO or WT CD8^+^ T cells (lower chamber). The results showed that monocytes exhibited enhanced migration toward cKO CD8^+^ T cells (Figure 3H). Treatment with recombinant CCL5 (rmCCL5) further promoted monocyte migration in WT co‐cultures, whereas CCL5 neutralization markedly reduced chemotaxis in both groups (Figure 3I).

To determine the direct effect of *Socs1*‐deficient CD8^+^ T cells on macrophage polarization, we co‐cultured bone‐marrow‐derived macrophages (BMDMs) with splenic CD8^+^ T cells from WT or cKO mice (Figure 3J). The presence of cKO CD8^+^ T cells induced a strong M1‐like phenotype in BMDMs, reflected by a marked increase in the expression of CD80, CD86, and MHC Class II (Figure 3K). Moreover, we measured higher concentrations of TNF‐α in the supernatants from the cKO CD8^+^ T cell co‐cultures system (Figure S3E). Taken together, these data demonstrate that *Socs1* deficiency in T cells orchestrates a profound shift in the macrophage compartment, promoting the accumulation and polarization of pro‐inflammatory, M1‐like macrophages equipped for enhanced interaction with CD8^+^ T cells.

Clinically, CCR1 expression in peripheral monocytes was consistently higher in aGVHD patients than in non‐aGVHD patients post‐transplantation (Figure S3F), and CD8^+^ T cell‐monocyte interactions were elevated in aGVHD patients compared to healthy controls (Figure S3G). Additionally, in dataset GSE229733 [37], monocytes from aGVHD patients exhibited increased M1‐associated gene expression, while non‐aGVHD patients showed higher M2 gene signatures (Figure S3H). Together, these findings suggest that *Socs1* deficiency in CD8^+^ T cells promotes monocytes polarization toward an inflammatory M1 phenotype, and heightened CD8^+^ T cell‐monocyte interaction may contribute to aGVHD development.

### 2.4 *Socs1*‐Deficient T Cells Infiltrate and Reshape the Intestinal Immune Microenvironment to Exacerbate aGVHD Severity

**FIGURE 4:**
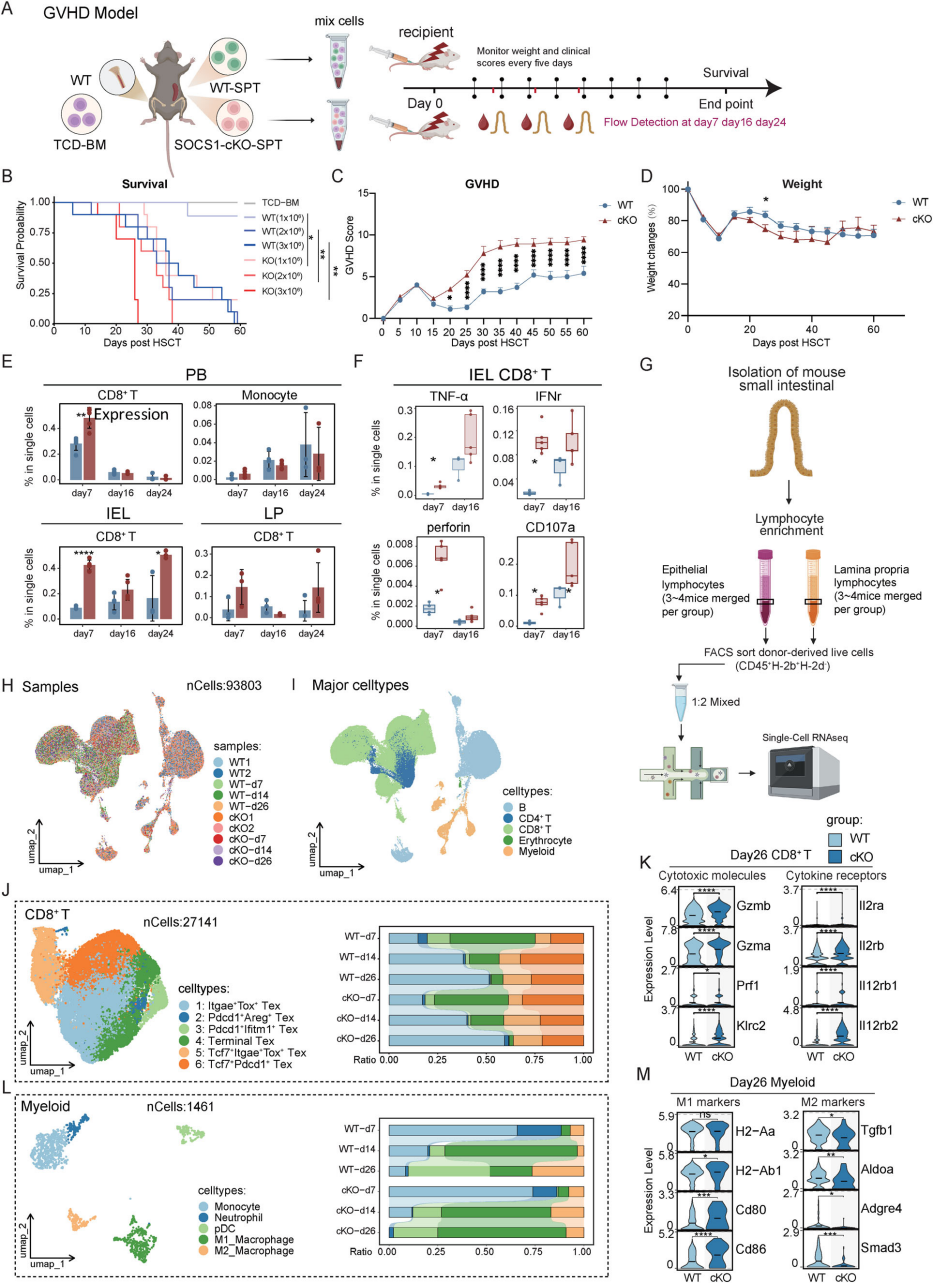
Evolution of small intestinal immune cell composition following transplantation of *Socs1* cKO CD8^+^T cells. (A) Experimental schematic. Lethally irradiated BALB/c recipient mice were transplanted with splenic T cells from WT mice (WT group) or cKO mice (cKO group), along with 5 × 10^6^ TCD‐BM cells from WT mice. Survival was monitored daily. Body weight and GVHD score were assessed every five days. Immune cells in PB and small intestine from WT and cKO groups were assessed on Day 7, Day 16, and Day 24 by flow cytometry. (B) Survival analysis of recipients transplanted with 1 × 10^6^, 2 × 10^6,^ or 3 × 10^6^ splenic T cells from WT or cKO mice (n = 10 mice per group). A control group received TCD‐BM only. Median survival times for cKO groups were 36 (1 × 10^6^), 33 (2 × 10^6^), and 26 days (3 × 10^6^), respectively. In the corresponding WT groups, 9/10 mice in the 1 × 10^6^ group survived to the end of the observation period, with median survival times of 35 days (2 × 10^6^), and 42.5 days (3 × 10^6^). Data were pooled from two independent experiments (n=10 mice/group). (C‐D) GVHD score (C) and body weight changes (D) in WT and cKO recipients transplanted with 1 × 10^6^ splenic T cells. (E) Proportions of CD8^+^ T cells and monocytes in PB, and proportions of CD8^+^ T cells in the IEL and LP on Day 7, 16, and 24 post‐transplantation. (F) Boxplots showing the expression of TNF‐α, IFN‐γ, perforin, and CD107a in CD8^+^ T cells in IEL by flow cytometry on Day 7 and 16 post‐transplantation. (G) Experimental schematic of scRNA‐seq. Lymphocytes from IEL and LP were isolated and collected from 3‐4 mice, followed by FACS sorting for CD45^+^ donor‐derived (H‐2^b+^ H‐2^d−^) cells respectively. Sorted cells from IEL and LP were mixed in a 1:2 ratio and subsequently subjected to scRNA‐seq. (H‐I) UMAP plot of all immune cells colored by samples (H) or annotated subsets (I). (J) UMAP plot showing annotated CD8^+^T cell subsets (left), with barplot illustrating the proportion of each annotated subset in recipient intestines at indicated time points between WT and cKO groups (right). (K) Violin plots comparing the expression of cytotoxic molecules and cytokine receptor genes in CD8^+^ T cells on Day 26 post‐transplantation between WT and cKO groups. (L) UMAP plot showing annotated myeloid cell subsets (left), with barplot illustrating the proportion of each annotated subset in recipient intestines at indicated time points between WT and cKO groups (right). (M) Violin plots comparing expression of M1‐ and M2‐associated marker genes in myeloid cells on Day 26 post‐transplantation between WT and cKO groups. Data represent three independent experiments. *P* values were determined using chi‐squared test (B) or unpaired two‐tailed Student's t‐test (E, F) or two‐sided Wilcoxon rank‐sum test (K, M). Differences in GVHD scores and body weight between the WT and cKO groups at each time point were analyzed using multiple unpaired two‐tailed Student's *t*‐tests (C, D). Data represent mean ± SEM (C‐E). ^∗^ *p* <.05, ^∗∗^ *p* <.01, ^∗∗∗^ *p* <.001 and ^∗∗∗∗^ *p* <.0001.

To further explore the role of SOCS1 in regulating T cells during aGVHD, we isolated splenic T cells from WT or cKO mice and transplanted them, along with T cell‐depleted bone marrow (TCD‐BM) cells (5 × 10^6^) from WT mice, into lethally irradiated BALB/c recipients to establish an aGVHD model (Figure 4A). Survival analysis demonstrated that recipient mice receiving *Socs1*‐deficient (cKO) T cells experienced significantly accelerated and dose‐dependent mortality compared to those receiving WT cells (Figure 4B). This lethal GVHD phenotype was accompanied by significantly higher clinical GVHD scores, which emerged around Day 20 post‐HSCT (Figure 4C). This severe pathology was macroscopically evident; by Day 24, cKO recipients displayed extensive skin lesions and splenic atrophy, in stark contrast to the WT group (Figure S4A). In addition, the cKO group exhibited a trend toward impaired body weight recovery. Although this difference did not consistently reach statistical significance, the mean body weight of cKO recipients remained lower than that of the WT group throughout the later stages of the experiment (Figure 4D). Flow cytometry revealed that donor‐derived CD8^+^ T cells were more abundant in the peripheral blood of cKO recipients on Day 7, but declined in both groups from Day 16 to 24, while monocytes gradually increased in both WT and cKO groups (Figure 4E). In the intestinal intraepithelial lymphocytes (IEL) and lamina propria (LP), CD8^+^ T cells infiltration was consistently higher in cKO recipients throughout the course of aGVHD (Figure 4E; Figure S4B). Moreover, these infiltrating CD8^+^ T cells in the cKO group exhibited elevated expression of TNF‐α, IFN‐γ, perforin, and CD107a compared to the WT group (Figure 4F). These results suggest that *Socs1* deficiency in T cells promotes CD8^+^ T cells activation and intestinal infiltration, contributing to more severe GVHD pathology.

To investigate donor‐derived lymphocyte infiltration in the recipient GI tract during GVHD, we sorted CD45^+^ donor‐derived cells from IEL and LP, mixed them at a 1:2 ratio, and performed single‐cell RNA sequencing (Figure 4G). To better elucidate the dynamics of donor lymphocytes from the steady state to post‐transplantation, we also included single‐cell transcriptomes of CD45^+^ splenic cells from WT and cKO mice (Figure 4H; Figure 1A). Based on the expression of signature genes, five major cell populations were identified: B cells (*Pax5, Ms4a1, Cd79a, Ebf1*), CD4^+^ T cells (*Tnfsf4, Epas1, Ramp3, Cd4*), CD8^+^ T cells (*Cd8a, Cd8b1, Gzma, Cd7*), Erythrocytes (*Hba‐a1, Hbb‐bt, Hebp1*) and Myeloid cells (*Hp, Ifitm6, S100a8, S100a9, Cxcr2, Retnlg*) (Figure 4H,I; Figure S4C). Integration and mapping of CD8^+^ T cells revealed distinct subset distributions in the steady state versus post‐transplant intestinal infiltration. To scrutinize the dynamics of pathogenic CD8^+^ T cells within the target organ, we focused on those infiltrating the small intestine. These cells predominantly adopted an exhausted phenotype over the course of GVHD (Figure S4G). However, this exhausted T cell (Tex) compartment was highly heterogeneous and could be subclustered into distinct populations based on marker expression, including a terminally exhausted subset (Terminal Tex: Pdcd1^+^ Lag3^+^ Havcr2^+^ Ctla4^+^) and a tissue‐resident‐like subset (Itgae^+^ Tox^+^ Tex), among other populations (Figure 4J; Figure S4E‐F). A key finding was a progressive compositional shift over time: the proportion of Terminal Tex cells markedly declined from Day 7 to Day 26, while the Itgae^+^ Tox^+^ Tex population concurrently expanded. This transition from a terminally exhausted to a tissue‐resident exhausted state was significantly more pronounced in cKO recipients by Day 26 (Figure 4J). This phenotypic shift was underpinned by corresponding changes at the transcript level, with a progressive upregulation of *Itgae* and the exhaustion‐master‐regulator *Tox*, coupled with a significant downregulation of *Pdcd1* expression at later time points (Figure S4G).

Notably, CD8^+^ T cells from cKO recipients exhibited significantly higher expression of cytotoxic molecules (*Gzmb*, *Gzma*, *Prf1*, *Klrc2*) and cytokine receptors (*Il2ra*, *Il2rb1*, *Il12rb1*, *Il12rb2*) on Day 26 compared to those from WT recipients (Figure 4K). We further observed that CD8^+^ T cells from WT recipients in the small intestine at Day 7 post‐transplant exhibited elevated Ccl5 expression relative to the cKO recipients, potentially facilitating monocyte and macrophage recruitment [38] (Figure S4G). As expected, *Socs1* deficiency in T cells reshaped the local myeloid landscape in the recipient GI tract following infiltration during aGVHD (Figure 4L; Figure S4D). By Day 26, WT recipients exhibited a phenotypic shift from M1 to M2 macrophages, whereas cKO recipients maintained a higher proportion of M1 macrophages (Figure 4L). Transcriptomic analysis further confirmed increased expression of M1‐associated genes (*H2‐Aa*, *H2‐Ab1*, *Cd80*, *Cd86*) and decreased expression of M2 markers (*Tgfb1*, *Aldoa*, *Adgre4*, *Smad3*) in cKO recipients (Figure 4M), consistent with global M1/M2 scoring (Figure S4H‐I). These findings suggest that *Socs1*‐deficient T cells infiltrate and modulate the intestinal immune microenvironment, promoting inflammatory M1 polarization of myeloid cells and aggravating GVHD pathology.

### 2.5 *Socs1*‐Deficient T Cells Sustain Crypt‐Localized Inflammation and Exacerbate Intestinal Injury in aGVHD

**FIGURE 5:**
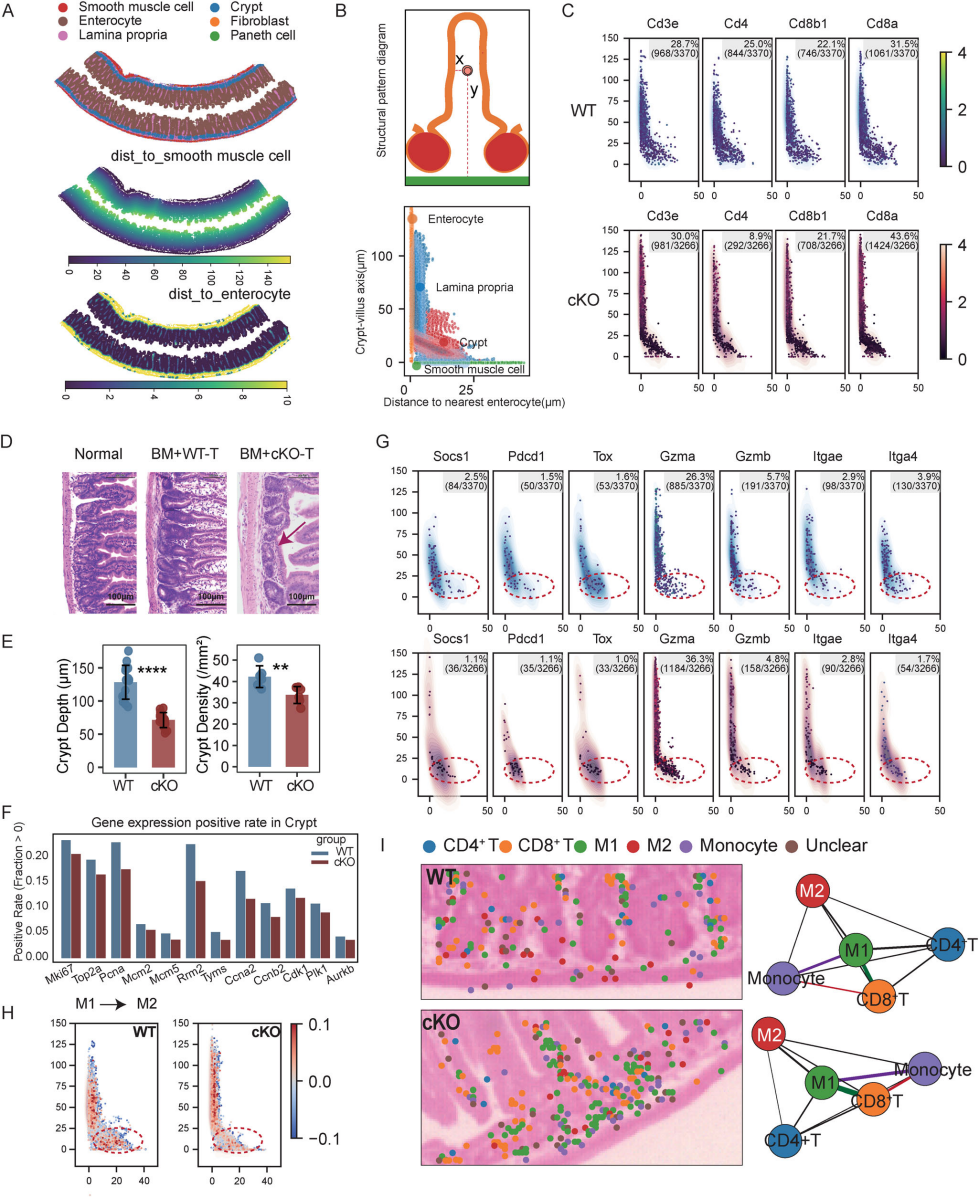
Spatial transcriptomics reveals the immune microenvironment of intestinal crypt systems on Day 24 post‐transplantation. At Day 24 post‐transplantation, small intestine samples from WT (Balb/c recipients transplanted with TCD‐BM from WT mice and Spleen T cells from WT mice) and cKO (Balb/c recipients transplanted with TCD‐BM from WT mice and Spleen T cells from cKO mice) recipients were collected and subjected to 10× HD spatial transcriptomics analysis. (A) Top, graph‐based clustering annotation results at 2 µm resolution. Middle, dotplot showing calculated distance from all spots to smooth muscle cells. Bottom, dotplot showing calculated distance from all spots to enterocytes. (B) Schematic diagram of villus structure (top); spot allocation plot (SAP) reconstruction of the cKO sample (bottom). (C) Display of all T cell‐associated spots on the SAP system. Colors represent the expression levels of the indicated genes at each spot. Only spots with gene expression > 0 are shown. The gene positive rate (number of positive spots / total number of T cell spots) is displayed in the upper‐right corner. (D) Representative images of H&E stained intestinal crypt architecture in normal mice, WT, and cKO transplanted recipients. The red arrow indicates crypt destruction. Scale bar, 100 µm. (E) Quantitative analysis of crypt depth (µm) and crypt area (mm^2^) in WT and cKO transplanted recipients. (F) Barplot showing the positive expression rates of cell cycle‐related genes in the crypt regions of WT and cKO groups. (G) Expression levels and distributions of indicated genes in T cell regions displayed on the SAP system. The dashed ellipse indicates the approximate location of the crypt. (H) Dotplot showing the distribution of M2 score minus M1 score in WT and cKO groups. M2 and M1 score of macrophage‐associated spots is assessed based on M2‐associated or M1‐associated markers (shown in Figure S5F). (I) Left, representative crypt regions showing the distribution of CD4^+^ T, CD8^+^ T, M1, M2, Monocyte, and Unclear spots. Right, cell‐cell spatial proximity network analysis among these five cell‐type spots across all crypt regions. *P* values were determined using an unpaired two‐tailed Student's t‐test (E). Data represent mean ± SEM (E). ^∗∗^ *p* <.01 and ^∗∗∗∗^ *p* <.0001.

To further characterize the spatial interactions between T cells and myeloid cells in the small intestine during GVHD, we performed high‐definition spatial transcriptomics (10x Genomics Visium HD) on the proximal jejunum section collected at Day 24 post‐transplantation. Intestinal tissues from WT and cKO recipient mice were co‐embedded on the same chip, with the top two sections representing the cKO group and the bottom two representing the WT group (Figure S5A–C). Graph‐based clustering identified six major spatial compartments: smooth muscle cells, crypts, enterocytes, fibroblasts, Paneth cells, and lamina propria. A spatial coordinate system named spot allocation plot (SAP) [39] was generated by calculating each spot's distance from smooth muscle cells (*Y*‐axis) and enterocytes (*X*‐axis) (Figure 5A,B; Figure S5B). Consistent with previous results, spatial analysis revealed increased CD8^+^ T cell infiltration in the intestines of cKO recipients compared to WT (Figure 4E; Figure 5C). Histological examination showed severe crypt destruction in cKO mice, indicating more extensive GVHD‐associated intestinal injury (Figure 5D,E; arrow; scale bar, 100 µm). In the crypt region, genes associated with cell proliferation (*Mki67*, *Top2a*, *Pcna*, *Mcm2*, *Mcm5*, *Rrm2, Tyms, Ccna2, Ccnb2, Cdk1, Plk1, Aurkb*) [40] were significantly downregulated in cKO recipients, suggesting impaired tissue regenerative capacity (Figure 5F). Moreover, inflammatory markers (*Gzma*, *Gzmb*, *Pdcd1*, *Tox*) and tissue‐resident markers (*Itgae*, *Itga4*) [39] were enriched in the crypt region of cKO recipients compared to WT (Figure 5G). These results indicate that in cKO recipients, CD8^+^ T cells preferentially localize to crypts, sustaining inflammation and disrupting intestinal recovery.

*Socs1*‐deficient CD8^+^ T cells also promoted M1 macrophage polarization within intestinal crypts, as indicated by a reduced transition from M1 to M2 macrophages in the cKO recipients (Figure 5H; Figure S5E). M2‐associated genes (*Tgfb1*, *Fabp4*) were highly expressed in WT crypts but significantly downregulated in cKO mice. Conversely, M1 markers (*Nfkb1*, *Rela*, *Fcgr1*) [41] were enriched in cKO crypts (Figure S5F). Spatial proximity analysis revealed dense clustering of CD8^+^ T cells, monocytes, and M1 macrophages within crypt regions in cKO mice (Figure 5I), suggesting enhanced local immune interactions. Together, these findings demonstrate that *Socs1*‐deficient CD8^+^ T cells preferentially localize to crypts, sustain a pro‐inflammatory microenvironment, drive M1 macrophage polarization, and impair tissue repair, thereby exacerbating intestinal GVHD.

### 2.6 CCL5‐Neutralizing Antibody, Ruxolitinib, and Maraviroc All Alleviate Intestinal GVHD in *Socs1*‐Deficient Models

**FIGURE 6:**
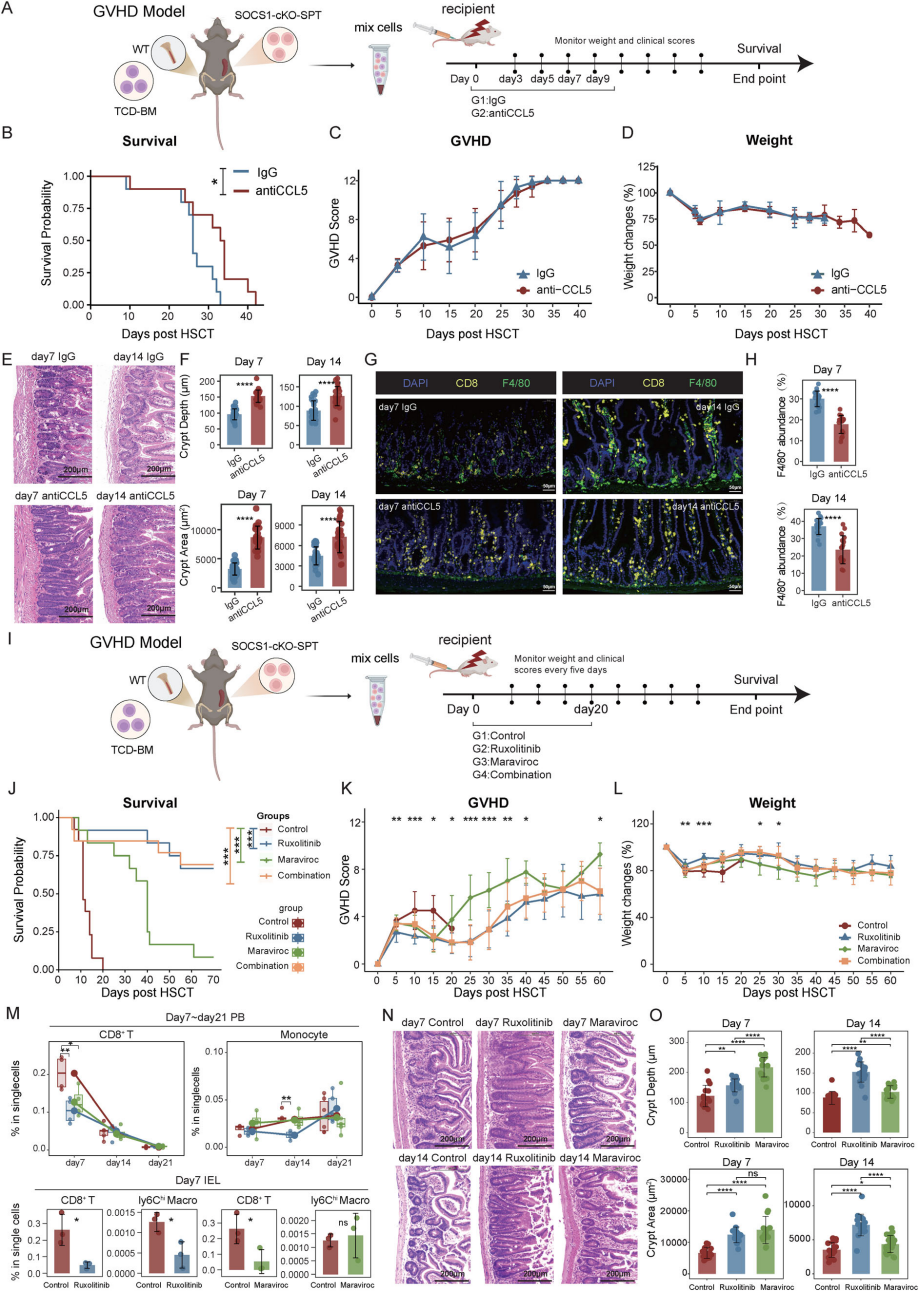
Inhibiting JAK/STAT signaling or CCL5 alleviates intestinal GVHD in *Socs1*‐deficient models. (A) Experimental schematic. Recipient mice were transplanted with 5 × 10^6^ T‐cell depleted bone marrow (TCD‐BM) cells from WT mice and 1 × 10^6^ splenic T cells from SOCS1‐cKO mice. Mice were treated with either control IgG or anti‐CCL5 antibody on Day 3, Day5, Day7 and Day9 post‐transplantation. (B–D) Survival curve (B), GVHD score (C), and body weight changes (D) of recipient mice treated with IgG or anti‐CCL5. Data are pooled from two independent experiments with a total of 8 mice per group. The median survival was 26 days in the IgG group versus 33.5 days in the anti‐CCL5 group. (E) Representative images of HE stained intestinal crypt architecture on Day 7 and Day 14 post‐transplantation in the IgG group and the anti‐CCL5 group. Scale bar, 200 µm. (F) Quantitative analysis of crypt depth (µm) and crypt area (µm^2^) on Day 7 and Day 14 post‐transplantation in the IgG group and anti‐CCL5 group. (G) Representative multiplex immunofluorescence images of the small intestine from mice treated with control IgG or anti‐CCL5 antibody, analyzed on Day 7 and 14 post‐transplantation. Sections were stained for CD8 (yellow), F4/80 (green), and DAPI (blue). Scale bar, 50 µm. (H) Quantification of the abundance of F4/80^+^ cells in the small intestine at the indicated time points. (I) Experimental schematic. BALB/c recipient mice were transplanted with 5 × 10^6^ TCD‐BM cells from WT mice and 1×10^6^ splenic T cells from cKO mice and treated with control vehicle, JAK/STAT inhibitor Ruxolitinib (30 mg/kg, i.g., BID), CCR5 antagonist Maraviroc (30 mg/kg, i.p., QD) or the combination from Day 1 to Day 20. Survival was monitored daily. Body weight and GVHD score were assessed every five days. (J‐L) Survival curve (J), GVHD score (K), and body weight changes (L) of the recipients in four groups. Each group included 12‐13 mice from two independent experiments. The median survival time in the control group was 11 days. At the observation endpoint (Day 70), 8/12 mice in the Ruxolitinib group, 1/12 mice in the Maraviroc group, and 9/13 mice in the Combination group remained alive. (M) Proportions of CD8^+^ T cells and monocytes in PB of recipients on Day 7, Day 14, and Day 21 (top, n = 3‐5 per group); and proportion of CD8+ T cells and Ly6Chi macrophage in the intestinal IEL of recipients on Day 7 (bottom, n = 3 per group) among indicated groups. (N) Representative images of HE stained intestinal crypt architecture on Day 7 and Day 14 post‐transplantation in the control, Ruxolitinib, and Maraviroc treatment groups. Scale bar, 200 µm. (O) Quantitative analysis of crypt depth (µm) and crypt area (µm^2^) on Day 7 and Day 14 post‐transplantation in the control, Ruxolitinib, and Maraviroc treatment groups. Data represent two independent experiments. *P* values were determined using chi‐squared test (B, J) or unpaired two‐tailed Student's t‐test (F, M, O). Weight loss and GVHD score (C‐D, K‐L) were analyzed by one‐way ANOVA. Data represent mean ± SEM (C‐D, F, K‐L, M, O). ^∗^ *p* <.05, ^∗∗^ *p* <.01, ^∗∗∗^ *p* <.001 and ^∗∗∗∗^ *p* <.0001.

Based on our previous findings that *Socs1* deficiency in T cells activates the CCL5 chemokine axis through JAK/STAT signaling, we designed a series of in vivo rescue experiments targeting CCL5, CCR5, and JAK1/2. To first evaluate the role of CCL5, we established a GVHD model by transplanting the WT TCD‐BM together with splenic T cells from cKO mice into lethally irradiated BALB/c recipients. Mice were treated with either control IgG or a CCL5‐neutralizing antibody on Day 0, 3, 5, 7 and 9 after transplantation (Figure 6A). Blockade of CCL5 conferred a significant survival benefit, extending median survival from 26 days in the IgG group to 33.5 days in the anti‐CCL5 group (Figure 6B). Despite this improvement, no significant differences were observed in overall GVHD clinical scores or body weight loss between the two groups (Figure 6C,D). Notably, histopathological analysis revealed a profound protective effect in the intestine. At Day 7 and Day 14 post‐transplantation, IgG‐treated mice exhibited severe crypt atrophy, with significantly reduced crypt depth and area. In contrast, anti‐CCL5 treatment largely preserved intestinal crypt architecture (Figure 6E,F). Consistent with these findings, multiplex immunohistochemistry of the small intestine at Day 7 and Day 14 revealed dense accumulation of F4/80^+^ macrophages surrounding the intestinal crypts in IgG‐treated mice. This macrophage infiltration was markedly reduced following CCL5 neutralization (Figure 6G,H), indicating that blockade of the CCL5 axis disrupts pathological macrophage recruitment and ameliorates intestinal injury.

To explore more effective therapeutic strategies, we utilized the same GVHD model to evaluate the efficacy of the clinically approved drugs Ruxolitinib (JAK1/2 inhibitor) and Maraviroc (CCR5 antagonist). Recipient mice were randomized into four treatment groups: vehicle control, Ruxolitinib, Maraviroc, or a combination of both drugs (Figure 6I). Treatments were administered daily from Day 1 toDay 20 post‐transplantation. As expected, the control group developed lethal acute GVHD, characterized by rapid mortality with all mice succumbing by Day 20 (Figure 6J), accompanied by escalating GVHD clinical scores and progressive weight loss (Figure 6K,L). While treatment with Maraviroc provided a significant, albeit modest, survival advantage over the control group, it failed to durably control disease progression, as evidenced by persistently high GVHD scores and poor weight maintenance in the surviving animals. In sharp contrast, Ruxolitinib treatment robustly protected recipients from GVHD lethality, with over 66.67% of mice surviving long‐term (Figure 6J). This dramatic improvement in survival was directly correlated with a sustained and significant reduction in GVHD clinical scores and stable weight recovery post‐transplantation (Figure 6K,L). Notably, a combination of Maraviroc and Ruxolitinib offered no synergistic or additive benefit over Ruxolitinib monotherapy across all measured parameters, including survival, clinical score, and body weight. Flow cytometry revealed reduced peripheral CD8^+^ T cells and decreased IEL infiltration in both Maraviroc and Ruxolitinib treatment groups (Figure 6M; Figure S6A,B). Monocyte and Ly6C^hi^ macrophage, which derived from Ly6C^hi^ monocytes, frequencies declined significantly with Ruxolitinib, but remained unchanged with Maraviroc (Figure 6M; Figure S6A,B), suggesting that Ruxolitinib targets both CD8^+^ T cells and myeloid cells, whereas Maraviroc primarily suppresses CD8^+^ T cell migration. By Day 60, immune cell profiles in Ruxolitinib‐treated mice resembled those of healthy controls (Figure S6C), indicating restoration of intestinal immune homeostasis. Histological analysis showed severe crypt damage in control mice by Day 7, including distortion, atrophy, and misalignment with the muscularis layer (Figure 6N). In contrast, Ruxolitinib and Maraviroc preserved crypt architecture, though by Day 14, crypt atrophy progressed in controls and emerged in Maraviroc‐treated mice, while Ruxolitinib‐treated mice maintained structural integrity (Figure 6O). These findings suggest that inhibition of the JAK/STAT/CCL5 axis, particularly via Ruxolitinib, prevents CD8^+^ T cell infiltration, preserves crypt architecture, and mitigates intestinal aGVHD damage in *Socs1*‐deficient models.

To determine the impact of JAK/STAT inhibition on the graft‐versus‐leukemia (GVL) effect, we established a murine GVL model by transplanting TCD‐BM from WT mice together with splenic T cells from either WT or *Socs1*‐cKO mice, and combined with GFP‐expressing AML‐ETO leukemia cells [42, 43, 44] (Figure S6D). Our results demonstrate that *Socs1*‐cKO T cells exert superior anti‐leukemia activity compared with WT controls (Figure S6E, H‐I, K). However, despite the enhanced GVL effect, recipient mice in the Socs1‐cKO group experienced reduced overall survival. Subsequently, to evaluate the therapeutic effects of Ruxolitinib, both WT and Socs1‐cKO GVL model mice were treated with Ruxolitinib continuously from Day 1 to Day 20 post‐transplantation, as detailed in Figure S6D. The treatment significantly alleviated GVHD scores and prevented weight loss, especially before Day 20 (Figure S6F, G). Although ruxolitinib partially attenuated GVL activity in both WT and Socs1‐cKO groups (Figure S6H–J). Ruxolitinib treatment significantly decreased the percentage of activated CD69^+^ CD8^+^ T cells in both genotypes (Figure S6J). Despite this, treatment still led to a significant extension of median survival in both WT and Socs1‐cKO groups (Figure S6E). Together, our data indicate that early pharmacological intervention with Ruxolitinib is required to temporally decouple GVHD toxicity from the therapeutic GVL effect of *Socs1*‐deficient CD8^+^ T cells, ultimately enabling tumor clearance and long‐term survival.

### 2.7 High *SOCS1* Expression in CD8^+^ T Cells Correlates with Reduced aGVHD Incidence following Allo‐HSCT

**FIGURE 7:**
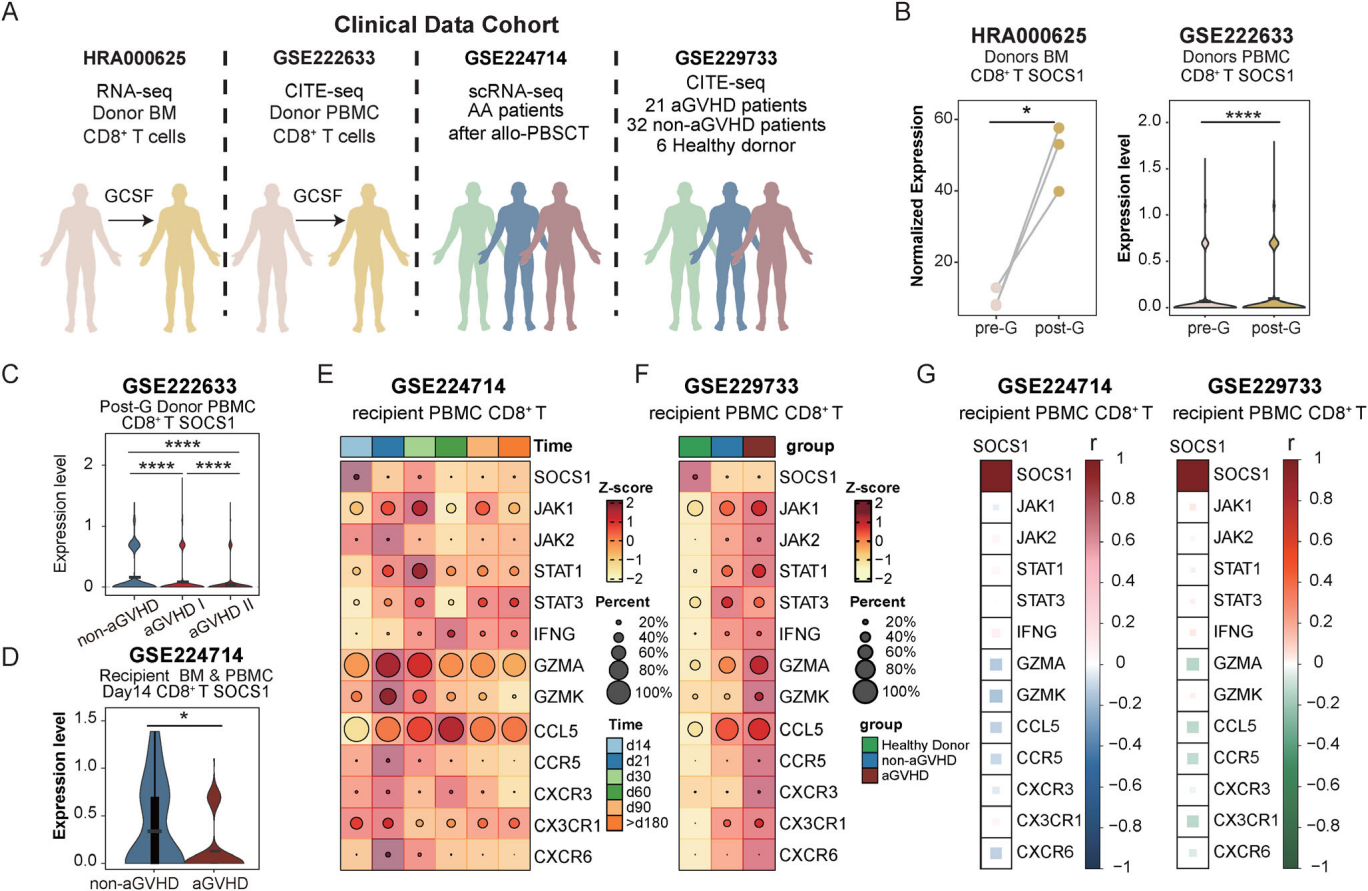
High *SOCS1* expression in CD8^+^ T cells correlates with reduced aGVHD incidence following allo‐HSCT. (A) Published sequencing datasets from clinical samples for reanalysis in this study. (B) Paired dot plot (left, GSE222633 dataset) and violin plot (right, GSE222633 dataset) showing SOCS1 expression in donor CD8^+^ T cells before and after G‐CSF administration. (C) Violin plot showing SOCS1 expression in CD8^+^ T cells in donor allograft post G‐CSF treatment among related recipients with non‐aGVHD, aGVHD I, and aGVHD II (GSE222633 dataset). (D) Violin plot showing SOCS1 expression in the CD8^+^ T cells from BM and PB on Day 14 post‐transplantation in non‐aGVHD and aGVHD recipients (GSE224714 dataset). (E) Heatmap demonstrating time‐course expression pattern of SOCS1‐related genes in CD8^+^ T cells from PB in recipients post‐transplantation (GSE224714 dataset). (F) Heatmap showing the expression pattern of SOCS1‐related genes in CD8^+^ T cells from Healthy donor, non‐aGVHD recipients, and aGVHD recipients (GSE229733 dataset). (G) Corrplots showing the Pearson correlation coefficients between SOCS1 and SOCS1‐related genes (GSE224714 and GSE229733 datasets). *P* values were determined using paired two‐tailed Student's *t*‐test (B‐left) or two‐sided Wilcoxon rank‐sum test (B‐right, C‐D). ^∗^ *p* <.05 and ^∗∗∗∗^ *p* <.0001.

Our murine model showed that *Socs1*‐deficient CD8^+^ T cells became activated, and the small intestine sustained a pro‐inflammatory microenvironment during GVHD. To validate these findings clinically, we analyzed four available transplantation‐related sequencing datasets (HRA000625 [21], GSE222633 [45], GSE224714 [27], GSE229733 [37]; Figure 7A). *SOCS1* expression in CD8^+^ T cells was elevated in both bone marrow and peripheral grafts following G‐CSF mobilization (Figure 7B), consistent with our previous study [21]. Notably, *SOCS1* expression in graft‐derived CD8^+^ T cells negatively correlated with aGVHD severity (Figure 7C), and lower *SOCS1* levels were associated with aGVHD onset (Figure 7D; Figure S7A,B). In line with our murine data, reduced *SOCS1* expression in CD8^+^ T cells correlated with JAK/STAT pathway activation and upregulation of the chemokine‐receptor axis (e.g., *CCL5, CCR5, CXCR3, CXCR*6) (Figure 7E–G). Although *SOCS1* expression was initially elevated in grafts, it declined after transplantation, especially in recipients who developed aGVHD, where the decrease was accompanied by heightened JAK/STAT and chemokine signaling (Figure 7E,F). Furthermore, aGVHD patients showed increased pro‐inflammatory monocyte populations, echoing findings from our mouse models (Figure S7C–F). Together, these results suggest that low *SOCS1* expression in CD8^+^ T cells and accumulation of pro‐inflammatory monocytes are associated with aGVHD development following allo‐HSCT. *SOCS1* expression in graft‐derived T cells may serve as a predictive marker for aGVHD risk.

## 3 Discussion

Our study identifies SOCS1 as a critical intrinsic regulator that constrains T cell‐mediated intestinal injury during aGVHD. Loss of *Socs1* in the donor T‐cell compartment resulted in broad activation of multiple T cell subsets, including CD4^+^ T cells [23], CD8^+^ T cells, and NKT cells, with CD8^+^ T cells exhibiting the most pronounced pathogenic phenotype. This intrinsic T cell dysregulation established a pro‐inflammatory immune microenvironment in lymphoid organs under steady state. Following transplantation, *Socs1* deficiency further promoted T cell infiltration into the intestine and drove profound remodeling of the local immune microenvironment toward a sustained inflammatory state, resulting in exacerbated tissue damage. Mechanistically, *Socs1*‐deficient CD8^+^ T cells displayed enhanced activation of JAK/STAT‐dependent chemokine‐receptor signaling programs. Among these, CCL5 emerged as a prominently upregulated chemokine associated with increased recruitment of monocytes and macrophages to intestinal crypts, suggesting that dysregulated chemokine signaling represents a key downstream consequence of SOCS1 loss that amplifies pathogenic immune circuits during aGVHD.

In murine aGVHD models, T cell‐specific *Socs1* loss induced early and severe intestinal pathology, indicating that immune dysregulation begins rapidly after transplantation. Although *SOCS1* expression was initially upregulated in graft‐derived CD8^+^ T cells following G‐CSF mobilization in healthy individuals, it declined post‐transplantation—especially in patients who later developed aGVHD. This decline coincided with increased JAK/STAT and chemokine signaling, suggesting that early loss of SOCS1 may trigger immune remodeling and limit the window for effective intervention. These findings underscore the potential value of early prophylactic strategies targeting SOCS1‐regulated pathways.

Crucially, our analysis reveals that *Socs1* deficiency alters the trajectory of T cell exhaustion after transplantation, shifting the balance from terminally exhausted cells toward a pathogenic, tissue‐resident phenotype. While we observed a progressive decline in terminally exhausted T cells during disease progression, there was a selective expansion of an Itgae^+^ Tox^+^ subset that was significantly more pronounced in the *Socs1*‐cKO group. This aligns with recent evidence establishing TOX as a key factor in maintaining the survival of progenitor‐like exhausted T cells [46]. The persistence of this Itgae^+^Tox^+^ population suggests that these cells retain significant cytotoxic potential and contribute to sustained tissue injury despite their “exhausted” profile. Therefore, therapeutic strategies that deplete these Tox^hi^ progenitor‐like cells, or drive their irreversible transition into a terminal exhausted state, may hold greater potential for mitigating GVHD severity.

Therapeutically, both JAK1/2 inhibition with Ruxolitinib [22] and CCR5 blockade with Maraviroc, as well as neutralization of CCL5, effectively alleviated intestinal pathology in *Socs1*‐deficient GVHD models. Notably, CCL5‐neutralizing antibody treatment conferred robust protection against intestinal GVHD, which was associated with reduced macrophage accumulation. These findings suggest that CCL5‐dependent chemokine signaling represents an important downstream effector pathway contributing to intestinal immune pathology in the context of *Socs1*‐deficient T cell responses. However, given that SOCS1 deletion and CCL5 neutralization were applied systemically in our study, future investigations employing subset‐specific genetic approaches, such as targeted deletion of *Ccl5* in *Socs1*‐deficient CD8^+^ T cells, will be required to definitively determine the cellular sources of CCL5 and to dissect their respective contributions to macrophage recruitment and polarization during aGVHD. Notably, Ruxolitinib was more effective, likely due to its dual ability to inhibit CD8^+^ T cell infiltration and suppress M1 macrophage polarization, thereby preserving intestinal crypt architecture. While Ruxolitinib is well established as a treatment for steroid‐refractory aGVHD [47, 48, 49, 50, 51], our findings suggest it may also serve as a GVHD prophylactic agent. Current GVHD prophylaxis strategies mainly rely on inhibiting T cell proliferation, through blockade of DNA synthesis such as methotrexate (MTX) and mycophenolate mofetil (MMF), or suppressing calcineurin signaling to prevent T cell activation, including cyclosporine A (CsA) and tacrolimus (FK506). T cell activation depends on three major signals: i) TCR‐mediated antigen recognition; ii) co‐stimulatory signaling (CD28, ICOS); and iii) cytokine‐mediated signaling. Conventional GVHD prophylaxis strategies predominantly target the first two pathways. However, T cells can be readily activated by inflammatory cytokines released following HSCT preconditioning, mediated by DAMPs and PAMPs. The JAK/STAT pathway acts as a central signaling hub for numerous cytokine families, including type I and II interleukins, interferons, IFN‐like cytokines, colony‐stimulating factors, as well as hormones and growth factors. Accordingly, our findings suggest that Ruxolitinib may serve as a complementary GVHD prophylaxis strategy by targeting cytokine‐dependent T‐cell activation. Supporting this, clinical studies have shown that incorporating Ruxolitinib into standard prophylaxis reduces aGVHD incidence in non‐malignant diseases such as aplastic anemia [52] and β‐thalassemia [53], as well as in myelofibrosis, which is classified as a malignant hematologic disorder [54]. Nevertheless, further clinical experience and prospective trials will be required to establish Ruxolitinib as a standard agent for GVHD prophylaxis.

Beyond allogeneic HSCT, these findings hold broader implications for assessing SOCS1 function in adoptive cell therapies (ACT) and immune checkpoint blockade. While genetic targeting of negative regulators like SOCS1 is an emerging strategy to enhance anti‐tumor potency [23], our data underscore the inherent risk of immunopathology associated with such unchecked activation. Based on the “decoupling” effect observed in our study, we propose that tunable JAK inhibition could conceptually serve as a “safety switch” for SOCS1‐edited T cell therapies, potentially managing cytokine‐associated toxicities while preserving superior effector function.

In addition to T cell‐mediated damage, our data indicate that accumulation of inflammatory macrophages and crypt architecture disruption are central to aGVHD severity in *Socs1*‐deficient models. In the WT group, donor‐derived lymphocytes preferentially migrated to the villi, while monocytes differentiated into M2 macrophages that supported tissue repair. In contrast, *Socs1*‐deficient models lacked this regenerative response. Due to limited sample size and lack of longitudinal spatial transcriptomics, the temporal dynamics of lymphocyte infiltration and the mechanisms by which *Socs1*‐deficient CD8^+^ T cells impair M2 polarization remain to be elucidated. Given that intestinal stem cells reside in crypts and are essential for epithelial renewal, and that epithelial injury is inevitable following conditioning regimens, promoting epithelial regeneration, including via IL‐22 [55] or TGF‐β [56, 57], and redirecting lymphocyte migration away from crypts may hold promise for mitigating gastrointestinal GVHD.

## 4 Materials and Methods

### 4.1 Mice

LckCre‐*Socs1* ^fl/fl^ (T cell‐specific Socs1‐cKO) and *Socs1* ^fl/fl^ (littermate control; WT) mice (sex‐ and age‐matched) were generated in a previous study [21]. All mice were maintained in the specific pathogen‐free animal facilit y of Peking University People's Hospital. All experiments were performed with approval according to the National Institutes of Health's Guide for the Care and Use of Laboratory Animals (2024PHE009).

### 4.2 Murine GVHD Model

Female WT or cKO mice (8–10 weeks old) were used as donors in the murine GVHD model. Age‐matched female BALB/c recipients were subjected to a total of 7 Gy total body irradiation, divided into two split doses of 3.5 Gy with a 4 h interval between the two irradiations. After 4 h, varying numbers (1 × 10^6^, 2 × 10^6^, or 3 × 10^6^) of purified splenic T cells from either cKO or WT donor mice were transplanted via tail vein injection. All recipient mice additionally received 5 × 10^6^ T cell‐depleted BM cells from WT donors to support hematopoietic reconstitution. Splenic T cells were isolated by negative selection using the Pan T Cell Isolation Kit II (Miltenyi, 130‐095‐130), achieving a purity of >95%. Bone marrow (BM) cells were depleted of T cells using CD90.2 Microbeads (Miltenyi, 130‐121‐278). Survival was monitored daily, and GVHD severity was evaluated every five days by assessing clinical scores based on six parameters: weight loss, hunching posture, activity level, diarrhea, fur texture changes, and skin lesions; each was scored on a scale from 0 to 2. Total scores were used to assess GVHD progression.

### 4.3 GVL Model and Ruxolitinib Treatment

Building upon the established GVHD model, 7 Gy lethally irradiated BALB/c recipients were infused on Day 0 with 5 × 10^6^ WT TCD‐BM cells together with 1 × 10^6^ splenic T cells isolated from either WT or cKO donor mice. To evaluate GVL activity, mice were co‐transplanted with 1 × 10^5^ GFP^+^ AML::ETO leukemia cells along with donor grafts. Beginning on Day 1 post‐transplantation, recipient mice underwent a 20‐day treatment regimen with either vehicle control or Ruxolitinib (Selleck, S2902). Ruxolitinib was administered by oral gavage (i.g.) at 30 mg/kg twice daily (BID). Following transplantation, mice were monitored daily for survival and evaluated every 5 days for body weight changes and clinical GVHD scores. Leukemia burden was indicated by measuring the frequency of GFP^+^ cells in peripheral blood, as measured by flow cytometry.

### 4.4 CCL5 Neutralization In Vivo

The GVHD model was established as described above. Briefly, BALB/c recipients were transplanted intravenously (i.v.) with 5 × 10^6^ WT TCD‐BM cells together with 1 × 10^6^ splenic T cells isolated from cKO mice. Recipient mice were treated with anti‐CCL5 neutralizing antibodies (10 µg/mouse; R&D Systems, AF478) or isotype control antibodies. Antibodies were diluted in PBS and administered i.v. on days 0, 3, 5, 7, and 9 post‐transplantation. Following transplantation, mice were monitored daily for survival and assessed every 5 days for body weight and clinical GVHD scores.

### 4.5 Drug Treatment In Vivo

Starting from Day 1 post‐transplantation, mice were randomly divided into four groups and received a 20‐day treatment regimen: vehicle control, Ruxolitinib, Maraviroc, and combination therapy. Ruxolitinib was administered at 30 mg/kg via oral gavage (i.g.) twice daily (BID), with administrations spaced at least 6 h apart. Ruxolitinib (Selleck, S2902) was prepared fresh daily as a 6 mg/mL suspension in corn oil with 1% DMSO (Solarbio, D8371). Maraviroc (Selleck, S2003) was administered at 30 mg/kg via intraperitoneal injection (i.p.) once daily (QD). The Maraviroc working solution (6 mg/mL) was prepared by diluting a 120 mg/mL stock in DMSO with a vehicle of 5% DMSO, 40% PEG300, 5% Tween80, and 50% ddH2O. The vehicle control group received the respective vehicle (Vehicle 1 via i.p. injection, Vehicle 2 via i.g.) following the same dosing schedule. All treatments were administered in a fixed volume of 100 µL per mouse.

### 4.6 Intestinal IEL/LP Lymphocyte Isolation

To isolate intestinal lymphocytes, small intestines from 8 to 12 week‐old mice were harvested, cleaned of mesentery, washed, and incubated in a DTT solution (Solarbio, D1070) for 10 min to remove mucus. For IEL isolation, the tissue was first digested for 20 min at 37°C in Digest Solution I containing 30 mm EDTA (Solarbio, E1170), and the crude IEL suspension was collected. The remaining tissue was then digested for 60 min at 37°C in Digest Solution II containing 100 U/mL Collagenase E (Sigma, C2139) to obtain the LP cell suspension. Both the collected IEL and LP cell suspensions were subsequently purified on a 40%/80% Percoll (Sigma, P1644) density gradient, centrifuged at 1000 x g for 20 min with the brake off. Lymphocytes were collected from the 40%/80% interface, passed through a 40 µm filter, and resuspended in Buffer 2 (RPMI 1640 supplemented with 2% heat‐inactivated FBS) for subsequent analysis.

### 4.7 H&E Staining

For morphological analysis of the small intestine, tissue samples were collected, fixed in 4% paraformaldehyde, and embedded in paraffin. Sections of 4–5 µm thickness were stained with Hematoxylin and Eosin (H&E) to visualize the intestinal architecture. Images of the stained sections were captured using an Olympus BX53. These images were then analyzed using ImageJ software to quantify crypt morphology. Crypt depth was measured in micrometers (µm) from the base of the crypt to the crypt‐villus junction. Crypt density was determined by counting the number of crypts within a defined tissue area and was expressed as the number of crypts per square millimeter (crypts/mm^2^). The crypt area was quantified by tracing the outer boundary of individual, well‐oriented crypts, with the final value expressed in square micrometers (µm^2^). For each animal, measurements from multiple non‐overlapping fields were averaged to obtain a representative score.

### 4.8 Immunofluorescence Detection

Small intestines of mice were harvested and fixed overnight in 4% paraformaldehyde. Fixed tissues were stained using the following primary antibodies: anti‐CD8 (1:4000; servicebio, GB15068), anti‐F4/80 (1:5000; servicebio, GB113373). Fluorescent images were acquired under standard immunofluorescence microscopy conditions.

### 4.9 Monocyte Chemotaxis Assay

Monocytes and CD8^+^ T cells were isolated from mice for use in tranwell migration assays. CD8^+^ T cells were purified from the spleens of WT and cKO mice by positive selection using CD8a (Ly‐2) MicroBeads (Miltenyi, 130‐117‐044). Monocytes were isolated from the bone marrow of WT mice via negative selection using the Monocyte Isolation Kit (Miltenyi, 130‐100‐629). Chemotaxis was assessed using 24‐well transwell plates with 8 µm pore size inserts (Corning, 3422). In each well, 2 × 10^5^ monocytes were seeded into the upper chamber in 200 µL of RPMI 1640 medium containing 1% FBS. The lower chambers contained 1 × 10^6^ CD8^+^ T cells in 800 µL of RPMI 1640 supplemented with 10% FBS and 1% penicillin‐streptomycin. To compare the intrinsic chemoattractant activity, monocytes were allowed to migrate toward CD8^+^ T cells from either WT or cKO mice for 2 and 12 h. To specifically investigate the role of CCL5, further 12‐h assays were conducted. In these assays, recombinant mouse CCL5 (R&D, 478‐MR) was added to lower chambers containing WT CD8^+^ T cells at a final concentration of 250 ng/mL. For neutralization experiments, CCL5 neutralizing antibody (R&D, AF478) at 0.75 µg/mL or a corresponding isotype control (R&D, AB‐108‐C) at 0.75 µg/mL was added to lower chambers containing either WT or cKO CD8^+^ T cells. After incubated at 37°C for 12h, cells that migrated to the lower chambers were collected, washed with PBS, and assessed using Cytek Northern Lights instrument. For experiments involving stimuli, migration was expressed as a ratio calculated by dividing the number of migrated cells in the treatment condition by that in the corresponding untreated control.

### 4.10 BMDM and Co‐Culture Assays

Murine bone marrow–derived macrophages (BMDMs) were generated from freshly isolated BM cells of WT C57BL/6 mice. Cells were cultured in Dulbecco's modified Eagle medium (DMEM; Gibco, C11995500BT) supplemented with 10% heat‐inactivated FBS and 20 ng/mL GM‐CSF (PeproTech, 315‐02‐50UG) for 6 days, with medium replacement every other day. After 24 h, non‐adherent cells were collected and replated at 1.5 × 10^6^ cells/mL in 6‐well plates. By Day 6, macrophage purity was confirmed to be >90% by flow cytometry using CD11b and F4/80 staining. To assess the role of CD8^+^ T cells in macrophage polarization, splenic CD8^+^ T cells were isolated on Day 5 from WT or cKO mice using anti‐CD8 Microbeads (Miltenyi, 130‐117‐044). Purified CD8^+^ T cells (1 × 10^5^ cells/well) were activated with CD3/CD28 Dynabeads (Thermo Fisher Scientific, 11452D) in the presence of 300 IU/mL IL‐2 in 48‐well plates (300 µL total volume). After 24 h, both conditioned medium (CM) and activated CD8^+^ T cells were harvested and co‐cultured with BMDMs. On Day 7, macrophages were collected, and analyzed by flow cytometry to evaluate polarization status.

### 4.11 Enzyme‐Linked Immunosorbent Assay (ELISA)

Cytokine levels were quantified using ELISA kits according to the manufacturers' instructions. The following kits were used: IL‐1β (Proteintech, KE10003), IL‐6 (Proteintech, KE10007), and TNF‐α (Proteintech, KE10002). The assay sensitivities were <7.1 pg/mL for IL‐1β, <15.6 pg/mL for IL‐6, and <7.8 pg/mL for TNF‐α.

### 4.12 Flow Cytometry

Single‐cell suspensions from peripheral blood, spleen, intestinal IEL/LP lymphocyte were prepared from recipient mice at the indicated time points post‐transplantation. Cells were washed with FACS buffer (PBS containing 2% FBS) and stained with a viability dye (Zombie NIR Fixable Viability Kit, Biolegend) and fluorochrome‐conjugated antibodies against surface markers for 30 min at 4°C. For intracellular staining of transcription factors, cells were subsequently fixed and permeabilized using either the BD Pharmingen Transcription Factor Buffer Set (BD Pharmingen, 562574) and then incubated with the respective intracellular antibodies. For intracellular cytokine analysis, cells were stimulated for 6 h at 37°C with phorbol 12‐myristate 13‐acetate (PMA, 50 ng/mL; Sigma–Aldrich, P8139) and ionomycin (1 µg/mL; Sigma–Aldrich, I3909). A fluorochrome‐conjugated anti‐CD107a antibody and GolgiPlug (BD Pharmingen, 555029) were added for the final 5 h of stimulation. After stimulation, cells were sequentially subjected to surface staining, fixation and permeabilization, and intracellular cytokine staining. Data were acquired and analyzed on a Cytek Northern Lights instrument. Details for all antibodies are provided in Data S5.

### 4.13 dUTP‐RNA‐Seq and Analysis

FACS‐sorted CD8^+^ T cells from WT and cKO mice were diluted to a concentration of 1 × 10^6^ cells/mL, and 500 000 cells were used per sample for total RNA extraction. Total RNA was extracted using RNeasy Mini kits (Qiagen, 74104) based on cell count. Poly(A) mRNA was isolated with NEBNext Oligo d(T)25 beads. First‐strand cDNA synthesis was conducted using the NEBNext First Strand Synthesis kit (NEB, E7490), followed by second‐strand synthesis with a dNTP mix containing dUTP to enable strand specificity. The resulting double‐stranded cDNA was purified using AMPure XP beads, end‐repaired, and adaptors were ligated using the NEBNext Ultra II RNA Library Prep Kit for Illumina kit (NEB, E7770). The uracil‐containing strand was selectively removed using the USER enzyme, ensuring strand specificity. The cDNA was then PCR‐amplified, and size‐selected for 300–500 bp fragments. Libraries were sequenced on an Illumina HiSeq platform using paired‐end 150 bp reads, allowing for comprehensive analysis of strand‐specific transcriptomic data.

Raw paired‐end sequencing reads were first assessed for quality using FastQC (v0.12.1). Adapters and low‐quality sequences were removed using trim_galore (v0.6.10) with a Phred quality score cutoff of 20 and a minimum read length of 20 bp (–quality 20 –length 20). The quality of the trimmed reads was subsequently re‐evaluated with FastQC. The cleaned reads were then aligned to the mouse reference genome (mm39) using HISAT2 (v2.2.1). The resulting alignments were sorted by coordinate and converted to BAM format using Samtools (v1.9). Alignment quality and statistics were evaluated using samtools flagstat. Finally, gene‐level expression counts were quantified from the paired‐end reads using featureCounts (v2.0.3) from the Subread package, based on the GENCODE vM34 gene annotation (‐g gene_id ‐p). The output was a raw count matrix for each sample, which was used for subsequent downstream analysis.

### 4.14 ATAC‐Seq and Analysis

For ATAC‐seq library preparation, fresh cells were lysed without freezing. Starting with 50 000 cells, they were pelleted at 500 g for 5 min at 4°C, then washed once with PBS. The cells were resuspended in lysis buffer containing 10 mm Tris‐HCl (pH 7.4), 10 mm NaCl, 3 mm MgCl2, and 0.1% Igepal CA‐630, and lysed for 10 min at 4°C with intermittent mixing. Nuclei were pelleted and resuspended in 35 µL DNase‐free water for reaction with 5× TTBL and TTE Mix V50, and fragmented at 37°C for 30 min. DNA was purified using the MinElute PCR Purification Kit or VAHTS DNA Clean Beads. For MinElute purification, the PCR product was mixed with Buffer PB, loaded onto the column, washed with Buffer PE, and eluted in ddH_2_O. Alternatively, beads were equilibrated, mixed with the sample, washed twice with 80% ethanol, and eluted in nuclease‐free water. For PCR enrichment, purified DNA was amplified using TAB, PPM, index primers, and TAE under specific cycling conditions. Post‐PCR, library fragments (100–1000 bp) were selected using an ethanol‐cleaned bead‐based method, washed twice, and eluted in ddH_2_O for sequencing. Libraries were quantified and stored at ‐20°C. Total amounts were confirmed to exceed 10 ng in volume greater than 10 µL for sequencing submission, with sample concentrations ranging from 65.2 to 92.8 ng/µL.

Raw ATAC‐seq reads were first trimmed for adapters and low‐quality bases using Trimmomatic (v0.39). The cleaned reads were then aligned to the reference genome using BWA‐MEM, and PCR duplicates were removed with samtools (v1.9). Peak calling was performed on high‐quality (MAPQ > 30), deduplicated reads using MACS 2(v2.2.9.1). The resulting peaks were filtered against a genomic blacklist and merged across all samples to generate a consensus peak set. Key quality control metrics, including fragment size distribution, TSS enrichment, and Fraction of Reads in Peaks (FRiP), were calculated for each sample and aggregated using MultiQC (v1.14). For transcription factor footprinting analysis, the TOBIAS (v0.16.1) suite was used to correct for Tn5 insertion bias (ATACorrect), calculate footprint scores, and identify transcription factor binding sites within accessible chromatin regions.

### 4.15 CUT&Tag and Analysis

FACS‐sorted CD8^+^ T cells from WT and cKO mice were diluted to a concentration of 1 × 10^6^ cells/mL and aliquoted as 200 µL suspensions into 2 mL EP tubes. These CD8^+^ T cells were then activated by stimulating with a cocktail of anti‐CD3 (1 µg/mL), anti‐CD28 (1 µg/mL), IL‐2 (10 ng/mL), and IFN‐α (50 ng/mL) for 30 min at 37°C with rotation. CUT&Tag was subsequently performed using the Hyperactive Universal CUT&Tag Assay Kit for Illumina (Vazyme Biotech, TD904). For this, primary antibodies (pSTAT1, STAT2, CTCF, H3K27ac, H3K4me3, and IgG) were added to 50,000 cells per reaction at a dilution of 1:50 and incubated overnight at 4°C. After washing twice with Dig‐wash buffer, a secondary antibody was added and incubated at room temperature for 60 min. Following another two washes with Dig‐wash buffer, a mixture of 2 µL pA/G‐Tnp Pro and 98 µL Dig‐300 buffer was added, and samples were incubated at room temperature for 1 h. After washing twice with Dig‐wash buffer, 50 µL of TTBL buffer was added for tagmentation at 37°C for 1 h. The reaction was stopped with 2 µL of 10% SDS and DNA Spike‐in, followed by incubation at 55°C for 10 min. After extraction with phenol‐chloroform and ethanol precipitation, PCR was performed to amplify the libraries according to the manual. All libraries were sequenced on an Illumina HiSeq platform (PE150). The resulting raw sequencing data were processed and analyzed using a computational pipeline identical to that described for the ATAC‐seq analysis, encompassing read trimming, alignment, duplicate removal, peak calling, and quality control. Details for all antibodies are provided in Table S5.

### 4.16 scRNAseq by 10× Genomics and Analysis

Single‐cell RNA‐seq libraries were prepared using the Chromium Single Cell 3ʹ Reagent Kits v3 (10x Genomics), following the manufacturer's instructions. FACS‐sorted CD45^+^ cells isolated from the spleens of WT and cKO mice were washed three times with 0.04% BSA in DPBS and resuspended to a concentration of 700–1200 cells/µL, ensuring a viability of ≥85%. Cells were captured in droplets to achieve a targeted cell recovery. Post reverse transcription, emulsions were broken, and Barcoded‐cDNA was purified using Dynabeads, followed by PCR amplification. The amplified cDNA was then used for constructing 3ʹ gene expression libraries. For library construction, 50 ng of amplified cDNA was fragmented, end‐repaired, and double‐size selected with SPRIselect beads. Sequencing was performed on a NovaSeq platform (Illumina) to generate 150 bp paired‐end reads. Count matrix was generated from the raw FASTQ files using cellranger count (v7.1.0) with the mm10 reference transcriptome (refdata‐gex‐mm10‐2020‐A).

### 4.17 scTCRseq by 10× Genomics and Analysis

Based on the cell counts and the recommended cell concentration, CD3^+^ cells isolated from the spleens of WT and cKO mice were immediately loaded onto a 10x Chromium Next GEM Chip K following the manufacturer's user guide (CG000331). Single‐cell RNA sequencing was performed using the Chromium Next GEM Single Cell 5'’ Gel Bead and Library Construction Kit, along with the Single Cell V(D)J Amplification Kit. After cDNA amplification and target enrichment, the quality of the post‐amplification cDNA, V(D)J‐enriched cDNA, and the final libraries were assessed using a Qubit Fluorometer (Thermo Scientific) and a Bioanalyzer 2100 (Agilent Technologies), respectively. The libraries were then sequenced on a NovaSeq X PLUS platform (Illumina) to generate high‐quality paired‐end reads for comprehensive gene expression and immune repertoire analysis.

### 4.18 10x Genomics Visium HD and Analysis

A 6‐µm‐thick FFPE section from a Day 24 mouse sample was processed using the Visium HD protocol (CG000685) with mouse‐specific reagents (PN‐1000676), utilizing a Cytassist instrument for analyte transfer and an Illumina NovaSeq 6000 for sequencing. Raw sequencing data were processed with spaceranger (v3.1.3) mkfastq and spaceranger count to generate spatial gene expression matrices at 2 and 8‐µm resolution. Key histological regions, including smooth muscle cells, crypts, and enterocytes, were then annotated using Loupe Browser. To quantify the spatial position of each spot, a Spot Allocation Plot was constructed by computing the Euclidean distance from each spot to the nearest annotated smooth muscle cell (*Y*‐axis) and enterocyte (*X*‐axis) using a KDTree‐based nearest neighbor algorithm.

### 4.19 Statistical Methods

Quantitative data were expressed as mean ± S.E.M or mean ± SD from a minimum of three biological replicates for animal experiments, unless otherwise noted. No data were excluded from the analyses. For comparisons between two experimental groups, statistical significance was assessed using a two‐sided Student's t‐test or a non‐parametric test where assumptions for parametric testing were not met. Unless otherwise indicated, statistical comparisons were performed against the designated reference group. Detailed information regarding the specific statistical tests applied, sample sizes (n), data presentation, and exact *p*‐values is provided in the corresponding figure legends. A *p*‐value of less than 0.05 was deemed significant. All statistical analyses were conducted using GraphPad Prism (v10) or R (v4.4.3).

## Author Contributions

Z.G.W. and H.D.G. conceived the study. Z.G.W., B.X.W, X.Y.J., Q.Q.H., F.Q.Z., S.F., Q.Z., J.R.Z., J.N.T., and X.Y.W. performed the experiments. Z.G.W. and B.X.W. analyzed the data. Z.G.W. and H.D.G. wrote the manuscript. X.J.H. and H.D.G. provided a critical review of the manuscript. X.J.H. and H.D.G. supervised the study. All authors reviewed and provided feedback on the manuscript. Z.G.W., B.X.W., X.Y.J. contributed equally to this work.

## Conflicts of Interest

The authors declare no conflict of interest.

## Supporting information




**Supporting File**: advs73975‐sup‐0001‐SuppMat.docx.

**Supporting File**: advs73975‐sup‐0002‐DataFile.zip.

## Data Availability

The raw and processed sequencing data reported in this paper (RNA‐seq, ATAC‐seq, CUT&Tag, scRNA‐seq, and Visium HD) have been deposited in the Genome Sequence Archive (GSA) database under the accession number GSA: CRA036176, CRA036177, CRA036179, CRA036745. All original code developed for the downstream analyzes is publicly available on GitHub (https://github.com/Portulaca666/SOCS1_associated_code.git). Any additional information, resources, and reagents are available from the Lead Contact, X.J.H. (huangxiaojun@bjmu.edu.cn), upon reasonable request.
