# Supplementary material for: Loss of SOCS1 in Donor T Cells Exacerbates Intestinal GVHD by Driving a Chemokine‐Dependent Pro‐Inflammatory Immune Microenvironment
Source: Adv Sci (Weinh). 2026 Jan 25;13(18):e13735. doi: 10.1002/advs.202513735 (PMC13042394; doi:10.1002/advs.202513735)
Supplement: Supplementary file 1 — Supporting File: advs73975‐sup‐0001‐SuppMat.docx. [file ADVS-13-e13735-s001.docx]

**Supplemental Figures:**


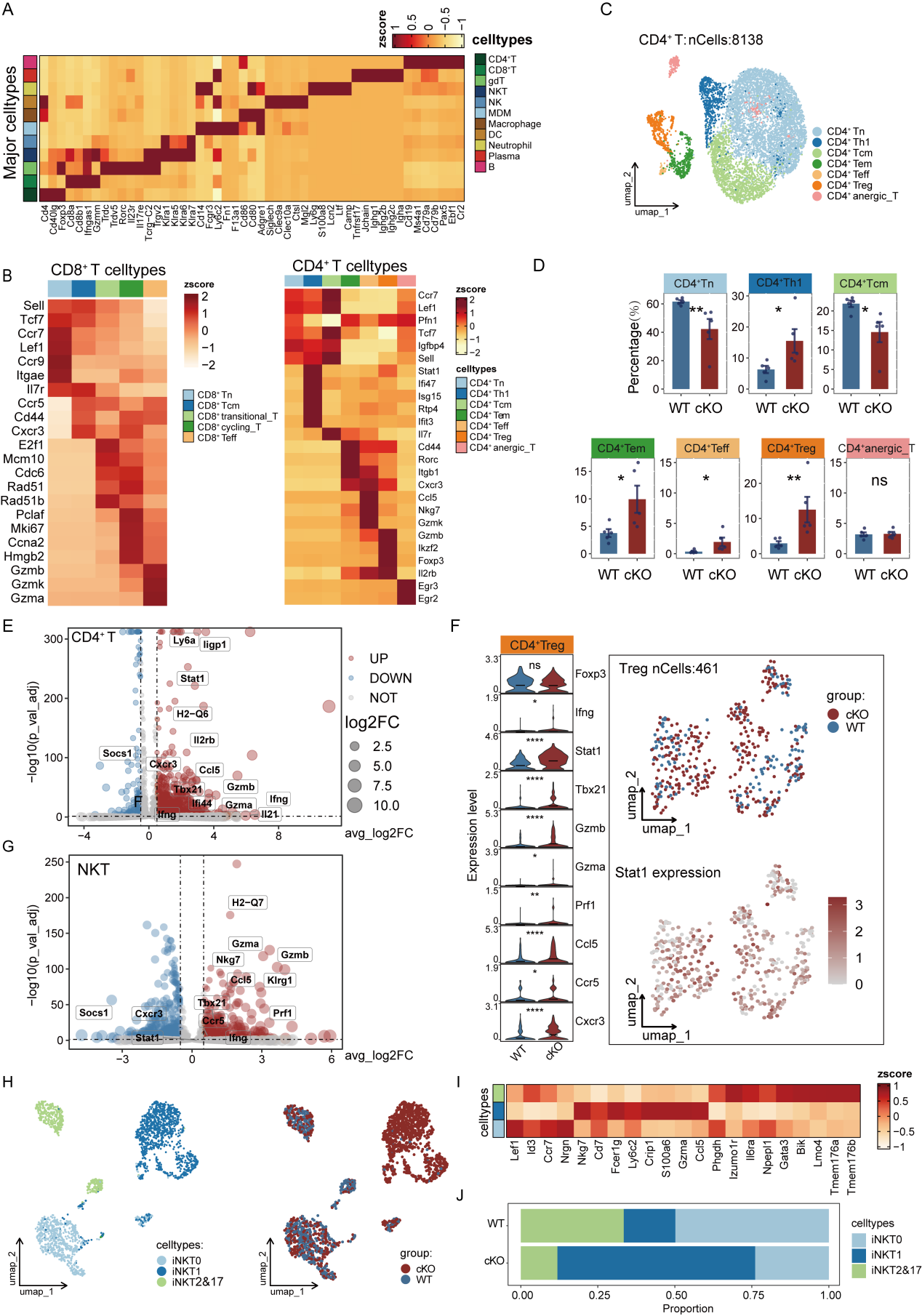


**Supplemental Figure 1. Annotation of immune cell subsets by single-cell RNA sequencing.**

1. Heatmap showing the expression of signature genes for each immune cell cluster, related to **Figure 1B**. Gene expression is scaled (z-score) by row. **(B)** Heatmap showing the expression of subset-specific marker genes for CD8^+^ T and CD4^+^ T cell clusters. Gene expression is scaled (z-score) by row. **(C)** UMAP plot of 8,138 CD4^+^ T cells colored by annotated T cell subsets. **(D)** Comparison of the proportions of indicated CD4^+^ T cell clusters between WT and cKO groups. **(E)** Volcano plot showing the results of the differential gene expression analysis of CD4^+^ T cells between the two groups. **(F)** Violin plots comparing the expression of several inflammatory genes in CD4^+^ Treg cells between the WT and cKO groups. Additionally, UMAP plots show the distribution of the two groups and the expression distribution of Stat1. **(G)** Volcano plot showing the results of the differential gene expression analysis of NKT cells between the two groups. **(H)** UMAP plot of NKT cells colored by annotated NKT cell subsets. **(I)** Heatmap showing the expression of subset-specific marker genes for NKT. **(J)** Bar plot showing the proportions of the NKT clusters between the two groups. *P* values were determined using two-sided Wilcoxon rank-sum test **(D, F)**. Data represent mean ± SEM **(D)**. ^∗^*P* < .05, ^∗∗^*P* < .01 and ^∗∗∗∗^*P* < .0001.

**
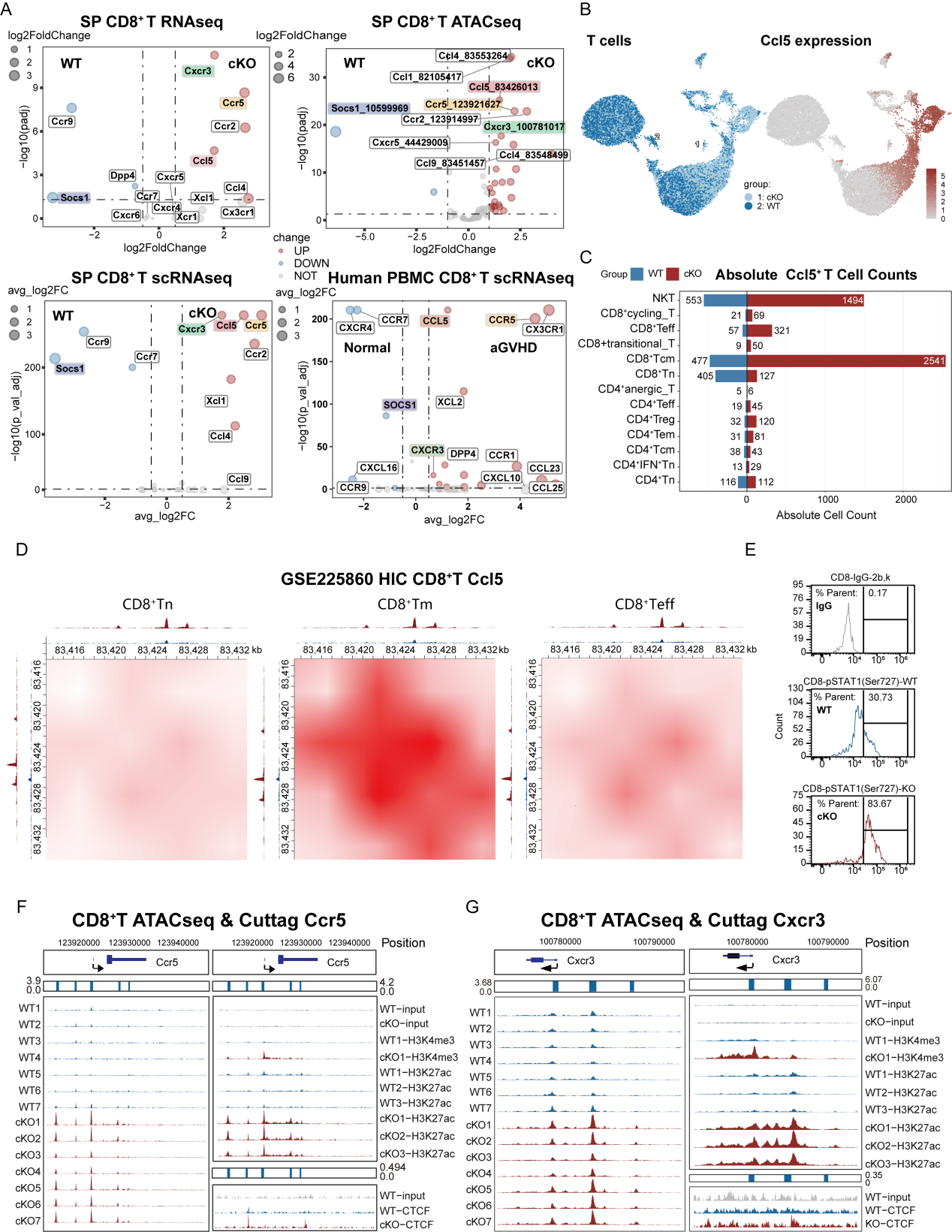
**

**Supplemental Figure 2. Upregulation of *Ccl5*, *Ccr5*, and *Cxcr3* in Socs1-deficient CD8^+^ T Cells.**

1. Volcano plots displaying the differential expression of chemokine family and *Socs1* in WT and cKO groups based on RNA-seq (top left panel), ATAC-seq (top right panel) and scRNA-seq data from **Figure 1** (bottom left panel), or showing the differential expression of chemokine family genes and *SOCS1* in aGVHD and Normal samples from CD8^+^ T cells extracted from GSE229733 dataset (bottom right panel). **(B)** UMAP visualization of CD3^+^ T cells split by group (WT vs. cKO), with feature plots showing Ccl5 expression across all CD3^+^ T cell subsets derived from scRNA-seq data. related to **Figure 1**. **(C)** Absolute counts of Ccl5^+^ T cells across all CD3^+^ T cell subsets as determined by scRNA-seq analysis. **(D)** Hi-C contact matrices from the GSE225860 dataset showing the chromatin interactions at the *Ccl5* locus in CD8^+^ Tn, Tm, and Teff cells. **(E)** Representative flow cytometry plots showing the expression of pSTAT1 (Ser727) in splenic CD8^+^ T cells from WT and cKO mice, related to **Figure 2H**. **(F-G)** ATAC-seq and CUT&Tag signal tracks at the *Ccr5* **(F)** and *Cxcr3* **(G)** loci in CD8^+^ T cells from WT and cKO mice. Left panels show ATAC-seq profiles indicating chromatin accessibility, and right panels show CUT&Tag data for H3K4me3, H3K27ac, input, and CTCF, reflecting chromatin state and regulatory features at each locus.

**
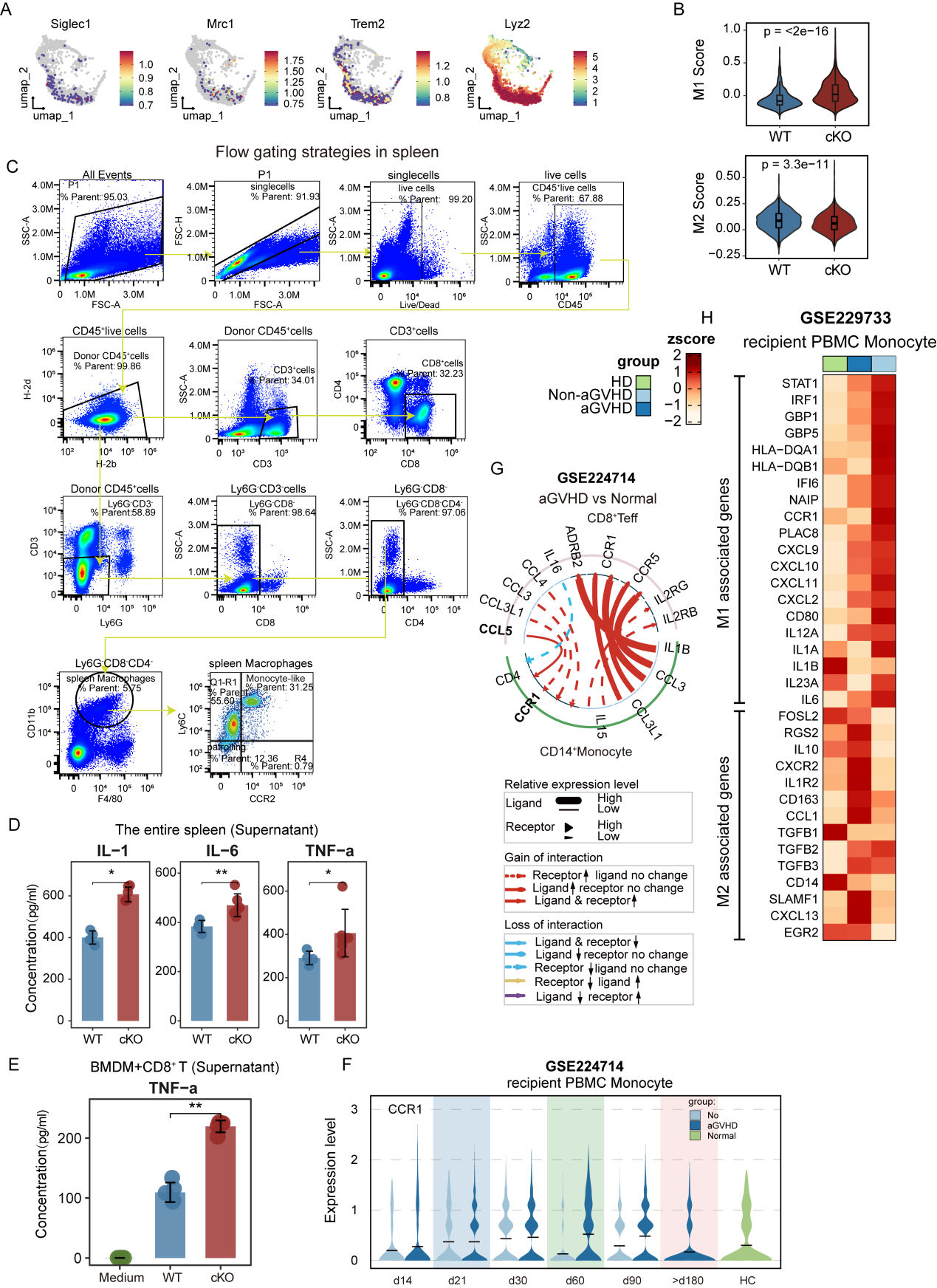
**

**Supplemental Figure 3. *Socs1*-deficient T cells Drive Monocyte Polarization Towards M1 Macrophages in Mice.**

**(A)** UMAP plots showing the expression of M2 macrophage-associated markers, related to **Figure 3A**. **(B)** Violin plots showing M1 and M2 signature scores in WT and cKO groups. **(C)** Gating strategy for macrophage populations in the spleen, related to **Figure 3E**. **(D)** Bar plot showing concentrations of IL-1, IL-6, and TNF-α in the supernatants of total splenocytes from WT and cKO mice, measured by ELISA. **(E)** Barplot showing the concentration of TNF-α in the supernatant of a co-culture system containing BMDMs and CD8⁺ T cells, quantified by ELISA. **(F)** Violin plots showing *CCR1* expression in PB from the Normal group and recipients with No-GVHD or aGVHD at the indicated time points post-transplantation (GSE246114 dataset). **(G)** iTALK-identified and visualized ligand-receptor interaction signals between CD8^+^ Teff and CD14^+^ Monocytes, illustrating differential intercellular communication patterns in aGVHD patients compared to Normal controls. **(H)** Heatmap showing the expression of M1- and M2-associated genes in blood monocytes across three groups (GSE229733 dataset). *P* values were determined using a two-sided Wilcoxon rank-sum test **(B, D-E)**. ^∗^*P* < .05 and ^∗∗^*P* < .01.


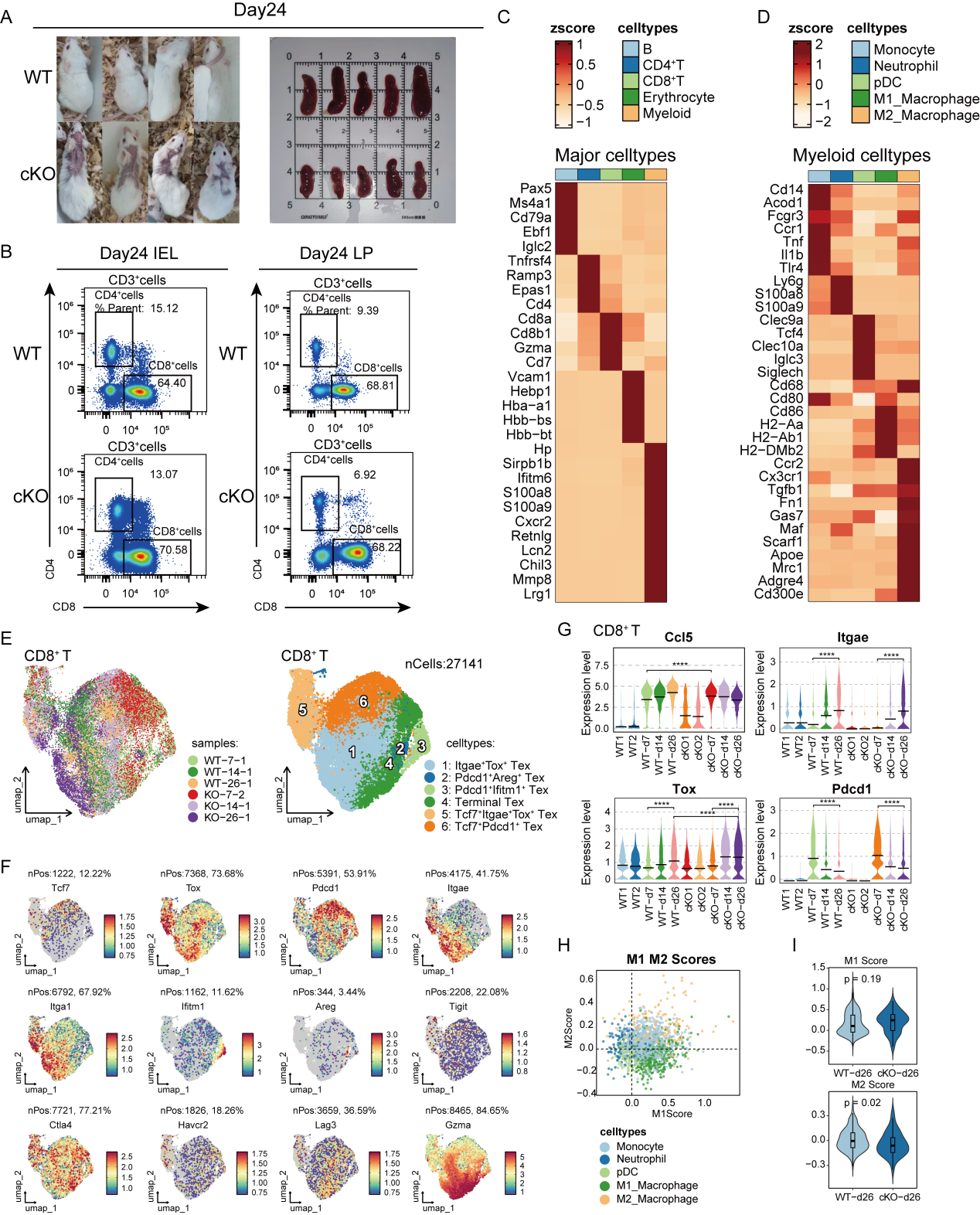


**Supplemental Figure 4. Comparison of immune cell types in small intestine between WT and cKO groups post-transplantation.**

1. Representative images of skin condition and spleen of the recipients from WT and cKO groups on Day 24 post-transplantation. **(B)** Representative flow cytometry plots showing CD8^+^ T cell gated in the IEL and LP from WT and cKO groups on Day 24 post-transplantation, related to **Figure 4E**. **(C)** Heatmap showing the expression of signature genes for each immune cell cluster, related to **Figure 4I**. **(D)** Heatmap showing the expression of signature genes for myeloid cell subsets, related to **Figure 4L**. **(E)** UMAP plot of all CD8^+^ T cells colored by samples (left) or annotated subsets (right), related to **Figure 4J**. **(F)** UMAP plots showing the expression of signature genes of each cluster. **(G)** Violin plots showing expression levels of *Ccl5*, *Itgae*, *Tox* and *Pdcd1* in CD8^+^ T cells across different samples. **(H-I)** Comparison of M1 and M2 scores between WT and cKO groups on Day 26 post-transplantation. **H** panel: scatter plot of M1 vs. M2 scores among myeloid cell types; **I** panel: violin plots comparing M1 and M2 scores between groups. P values were calculated using a two-sided Wilcoxon rank-sum test **(G, I)**. ^∗∗∗∗^*P* < .0001.

**
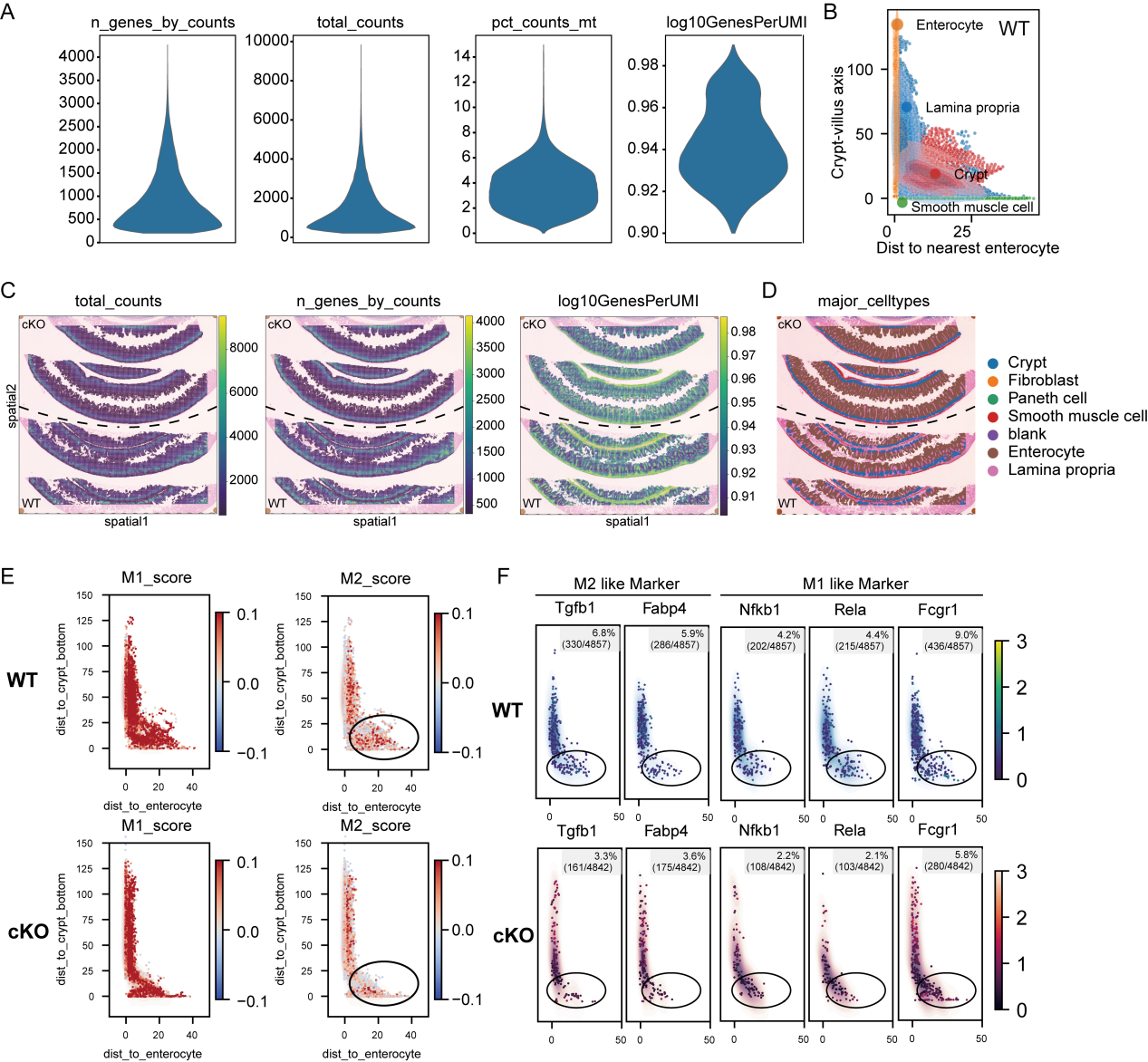
**

**Supplemental Figure 5. Quality control assessment and M1/M2 macrophage scoring in 10× HD spatial transcriptomics.**

**(A)** Violin plots showing quality control (QC) metrics at 2 μm resolution, including number of genes per spot (n_genes_by_counts), total transcript counts per spot (total_counts), percentage of mitochondrial transcript counts (pct_counts_mt), and log-transformed gene complexity (log10GenesPerUMI). **(B)** Spot Allocation Plot (SAP) of the WT sample, showing spot positions in relation to intestinal microanatomy including crypts, enterocytes, smooth muscle cells, and lamina propria, related to **Figure 5B**. **(C)** Spatial distribution maps of QC metrics across the full 10× HD spatial transcriptomics tissue section: total_counts, n_genes_by_counts, and log10GenesPerUMI. The upper half of the section corresponds to the KO sample, and the lower half corresponds to the WT sample. **(D)** Graph-based clustering and annotation of spatial compartments projected across the entire 10× HD spatial transcriptomics section, identifying regions such as crypts, fibroblast, Paneth cell, smooth muscle cell, blank, Enterocyte and Lamina propria. **(E)** M1 and M2 macrophage gene scores for all macrophage-associated spots in WT and KO groups. The black ellipse indicates the approximate location of the crypt. **(F)** Expression levels and distributions of selected marker genes for M2-like (*Tgfb1*, *Fabp4*) and M1-like (*Nfkb1*, *Rela*, *Fcgr1*) macrophages displayed on the SAP system.

**
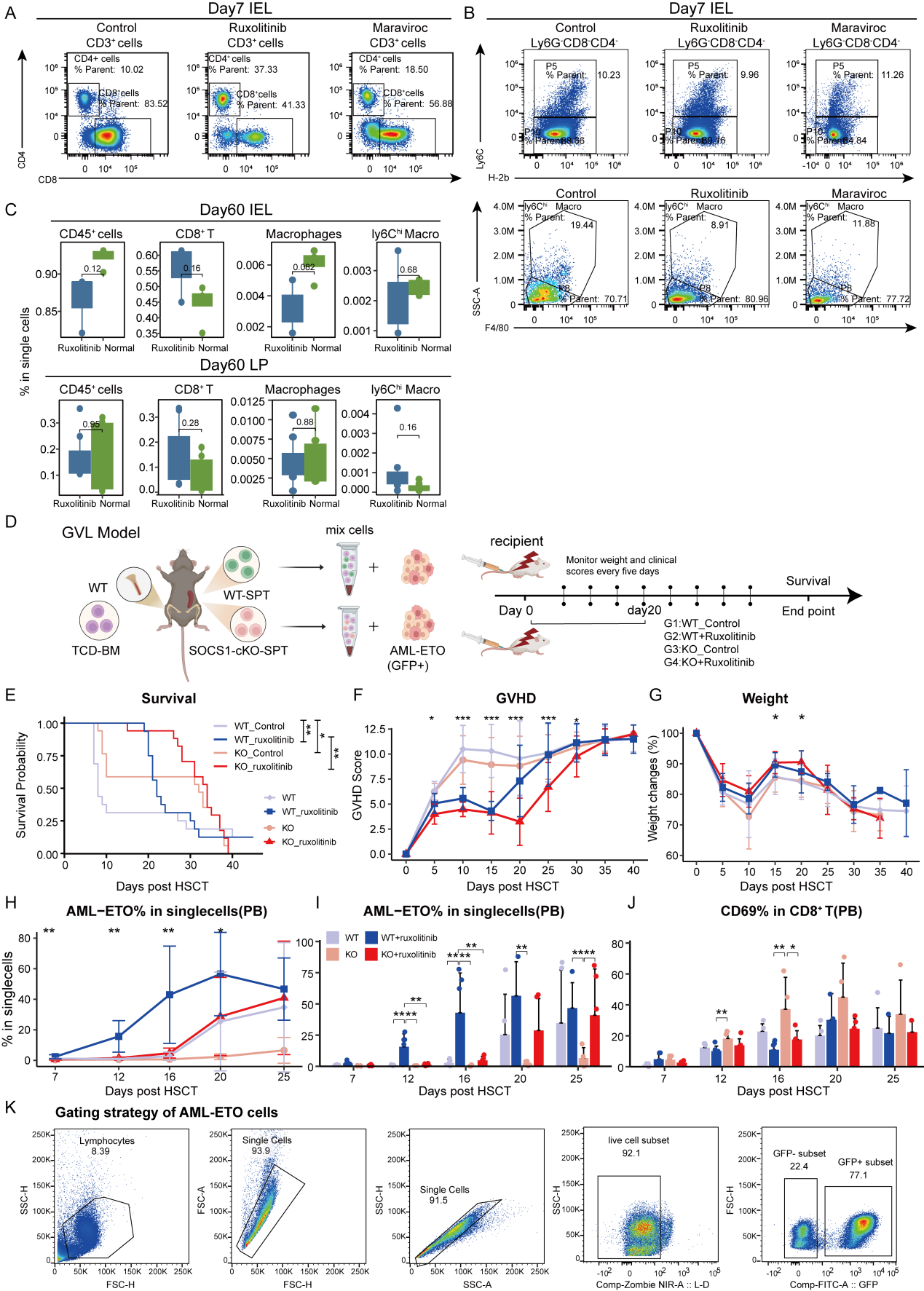
**

**Supplemental Figure 6. Inhibiting JAK/STAT signaling or CCL5 alleviates intestinal GVHD in *Socs1*-deficient models**

**(A-B)** Representative flow cytometry plots showing the proportion of CD8^+^ T cells **(A)** and Ly6C^hi^ macrophages **(B)** in IELs from Control, Ruxolitinib, and Maraviroc groups on Day 7 post-transplantation, related to **Figure 6E**. **(C)** Proportions of immune cell subsets in IELs and LP compartments from Control and Ruxolitinib groups on Day 60 post-transplantation. **(D)** Experimental schematic of the GVL model. Lethally irradiated recipient mice were transplanted with 5×10^6^ TCD-BM, along with 1×10^6^ splenic T cells from either WT or SOCS1-cKO donor mice, and co-injected with 1×10^5^ AML-ETO leukemia cells. Recipients were then treated with either vehicle control or the JAK1/2 inhibitor Ruxolitinib from Day 1 to Day 20. **(E-G)** Clinical outcomes of the four experimental groups. Survival curves **(E)**, GVHD scores **(F)**, and body weight changes **(G)** were monitored over time. In the WT groups, 2/10 mice survived in both the WT and WT_Ruxolitinib groups. In the cKO groups, the median survival times were 31.5 days and 34 days in the cKO and cKO_Ruxolitinib groups, respectively. **(H-I)** Longitudinal tracking of leukemia burden (AML-ETO%) in the peripheral blood (PB). **(J)** Longitudinal tracking of the percentage of CD69^+^ CD8^+^ T cells in PB. **(K)** Gating strategy for GFP^+^ AML-ETO cells in PB. Line and bar graphs represent mean ± SEM **(C, F-J).** P values were determined using chi-squared test **(E),** two-way ANOVA **(F-H)** or unpaired two-tailed Student’s t-test **(C, I-J)**. ^*^P < 0.05, ^**^P < 0.01 and ^***^P < 0.001.


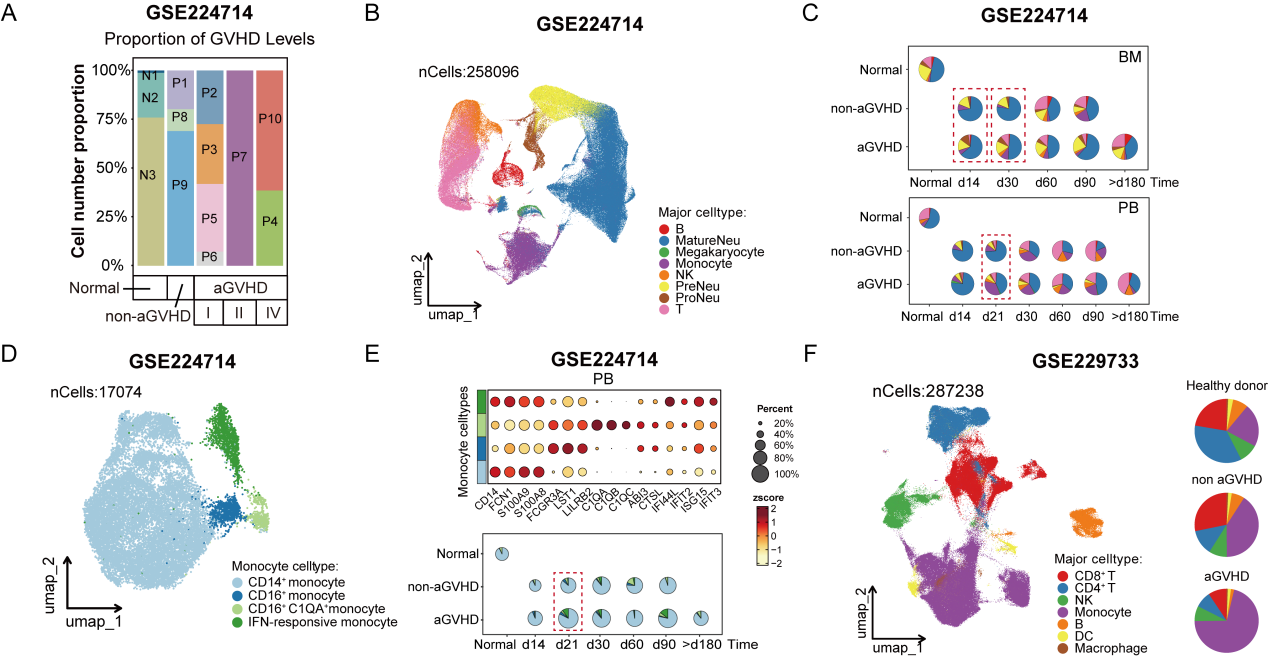


**Supplemental Figure 7. Assotiation between immune cell proportions and aGVHD occurrence in clinical sequencing datasets**

**(A)** Classification of clinical samples into Normal, aGVHD, and non-aGVHD groups based on clinical information from the GSE224714 dataset. **(B)** UMAP plot of 258,096 total nucleated cells (TNCs) colored by the annotated immune cell subsets from the GSE224714 dataset. **(C)** Pieplots depicting the composition of immune cells in the BM and PB of the Normal group and recipients with either non-aGVHD or aGVHD at the indicated time points post-transplantation. **(D)** UMAP plot of 17,074 monocytes colored by the annotated monocyte subsets from the GSE224714 dataset. **(E)** Feature gene expression profiles defining monocyte subsets (top), and the composition of monocyte subsets in the PB of different clinical groups at indicated time points post-transplantation (GSE224714 dataset). **(F)** UMAP plot of 287,238 immune cells colored by the annotated subsets (left), and pieplots showing the composition of immune cells in three clinical groups (GSE229733 dataset).

**Supplementary Data 1.** Chemotaxis-related differentially expressed genes in splenic Socs1-cKO versus WT CD8+ T cells (RNA-seq).

(Separate File)

**Supplementary Data 2.** Chemotaxis-associated differentially accessible regions (DARs) in splenic Socs1-cKO versus WT CD8+ T cells (ATAC-seq).

(Separate File)

**Supplementary Data 3.** Chemotaxis-related differentially expressed genes in splenic Socs1-cKO versus WT CD8+ T cells (scRNA-seq).

(Separate File)

**Supplementary Data 4.** Chemotaxis-related differentially expressed genes in peripheral blood CD8+ T cells from aGVHD patients versus Healthy Donors (scRNA-seq).

**Supplementary Data 5.** Antibody information

(Separate File)
